# Phylogeography Analysis Reveals Rabies Epidemiology, Evolution, and Transmission in the Philippines

**DOI:** 10.1093/molbev/msaf007

**Published:** 2025-02-12

**Authors:** Liang Zhang, Jeffrey Cruz, Yao Tian, Yuyang Wang, Jianfeng Jiang, Riva Marie Gonzales, Rachel R Azul, Rainelda C Dela Peña, Sheng Sun, Yan Liu, Tao Jiang, Liqun Fang, Changchun Tu, Wenjie Gong, Ye Feng

**Affiliations:** State Key Laboratory for Diagnosis and Treatment of Severe Zoonotic Infectious Diseases, Key Laboratory for Zoonosis Research of the Ministry of Education, College of Veterinary Medicine, Jilin University, Changchun, China; Changchun Veterinary Research Institute, Chinese Academy of Agricultural Sciences, State Key Laboratory of Pathogen and Biosecurity, Key Laboratory of Jilin Province for Zoonosis Prevention and Control, Changchun, China; Animal Disease Diagnosis and Reference Laboratory, Veterinary Laboratory Division, Department of Agriculture Bureau of Animal Industry, Quezon, Philippines; State Key Laboratory of Pathogen and Biosecurity, Academy of Military Medical Sciences, Beijing, China; Institute of Pathogen Biology, Chinese Academy of Medical Sciences and Peking Union Medical College, Beijing, China; State Key Laboratory for Diagnosis and Treatment of Severe Zoonotic Infectious Diseases, Key Laboratory for Zoonosis Research of the Ministry of Education, College of Veterinary Medicine, Jilin University, Changchun, China; Animal Disease Diagnosis and Reference Laboratory, Veterinary Laboratory Division, Department of Agriculture Bureau of Animal Industry, Quezon, Philippines; Animal Disease Diagnosis and Reference Laboratory, Veterinary Laboratory Division, Department of Agriculture Bureau of Animal Industry, Quezon, Philippines; Animal Disease Diagnosis and Reference Laboratory, Veterinary Laboratory Division, Department of Agriculture Bureau of Animal Industry, Quezon, Philippines; Changchun Veterinary Research Institute, Chinese Academy of Agricultural Sciences, State Key Laboratory of Pathogen and Biosecurity, Key Laboratory of Jilin Province for Zoonosis Prevention and Control, Changchun, China; Changchun Veterinary Research Institute, Chinese Academy of Agricultural Sciences, State Key Laboratory of Pathogen and Biosecurity, Key Laboratory of Jilin Province for Zoonosis Prevention and Control, Changchun, China; State Key Laboratory of Pathogen and Biosecurity, Academy of Military Medical Sciences, Beijing, China; State Key Laboratory of Pathogen and Biosecurity, Academy of Military Medical Sciences, Beijing, China; Changchun Veterinary Research Institute, Chinese Academy of Agricultural Sciences, State Key Laboratory of Pathogen and Biosecurity, Key Laboratory of Jilin Province for Zoonosis Prevention and Control, Changchun, China; Jiangsu Co-innovation Center for Prevention and Control of Important Animal Infectious Diseases and Zoonosis, Yangzhou University, Yangzhou, Jiangsu Province, China; State Key Laboratory for Diagnosis and Treatment of Severe Zoonotic Infectious Diseases, Key Laboratory for Zoonosis Research of the Ministry of Education, College of Veterinary Medicine, Jilin University, Changchun, China; Changchun Veterinary Research Institute, Chinese Academy of Agricultural Sciences, State Key Laboratory of Pathogen and Biosecurity, Key Laboratory of Jilin Province for Zoonosis Prevention and Control, Changchun, China; Changchun Veterinary Research Institute, Chinese Academy of Agricultural Sciences, State Key Laboratory of Pathogen and Biosecurity, Key Laboratory of Jilin Province for Zoonosis Prevention and Control, Changchun, China; State Key Laboratory of Pathogen and Biosecurity, Academy of Military Medical Sciences, Beijing, China

**Keywords:** rabies virus, Philippines, molecular epidemiology, transmission patterns, selection pressure, strategy

## Abstract

Rabies, caused by rabies virus, is a severe public health problem in the Philippines, where animal rabies epidemiology had been extensively investigated, but little is known about the national epidemiologic situations since 2010. Here, we report a 12-year nationwide animal rabies surveillance with systematic phylogenetic analysis, in which 353 whole genomes of rabies viruses collected from animal rabies cases between 2018 and 2022 were obtained. The phylogenetic and spatial–temporal evolutionary analyses showed that rabies viruses in the Philippines were exclusively classified into the SEA4 subclade within the Asian clade, but forming three major geographically specific lineages. Intra-island spread predominates the rabies transmission in three major island regions, while the inter-island transmission, between major island regions, is very limited, likely due to ocean barriers. Overall, our findings have provided the most comprehensive dataset on the infected animal species, geographic distribution, transmission dynamics, genetic diversity of rabies viruses, and transmission risk factors, thus established a basis to support WOAH-endorsed national control program for dog-mediated rabies in the Philippines.

## Introduction

Rabies, caused by the rabies virus (RABV), is a fatal zoonotic disease of humans and almost all warmblooded animals. It causes severe dysfunction of the central nervous system (Christie 1981). About 99% of human cases occur in developing countries, mainly in Asia and Africa (WHO 2024a). In the Philippines, rabies poses a significant public health issue, resulting in over 200 human deaths annually (WHO 2024b). Rabid dogs are the major sources of human rabies in the Philippines, as well as in other rabies-endemic countries (Dimaano et al. 2011). Previous studies have made significant contributions to understanding rabies in the Philippines, including the nationwide epidemiology researches based on partial gene before 2010 and subsequent regional studies using the whole genome sequencing (Nishizono et al. 2002; Saito et al. 2013; Tohma et al. 2014, 2016; Bacus et al. 2021; Capin et al. 2023). However, current studies on rabies epidemiology in the Philippines mainly focus on identifying factors associated with rabies spread, which include dog population density, climate variables, public education levels, and the availability of post-exposure prophylaxis (Tohma et al. 2016; Lachica et al. 2020; Swedberg et al. 2023). Reliable surveillance data for the country were absent (Capin et al. 2023), and a comprehensive nationwide analysis on the molecular epidemiology and genetic diversity for RABVs using whole genome sequencing remain unavailable in the Philippines.

Animal rabies surveillance has been implemented annually in the Philippines to control and possibly eliminate human rabies, and the country reached a new milestone when its official control program for dog-mediated rabies was endorsed by World Organization for Animal Health (WOAH) in May 2021. To better understand RABV evolution in the country, 353 full-length genome sequences of RABV strains along with epidemiological information were obtained during 2018 to 2022. We investigated the nationwide geographic distribution and genetic diversity of RABVs through phylogenetic and phylogeographic analyses in the Philippines, and then propose targeted measures for preventing and controlling rabies in the Philippines.

## Results

### Animal Rabies Situation in the Philippines

**Fig. 1.**
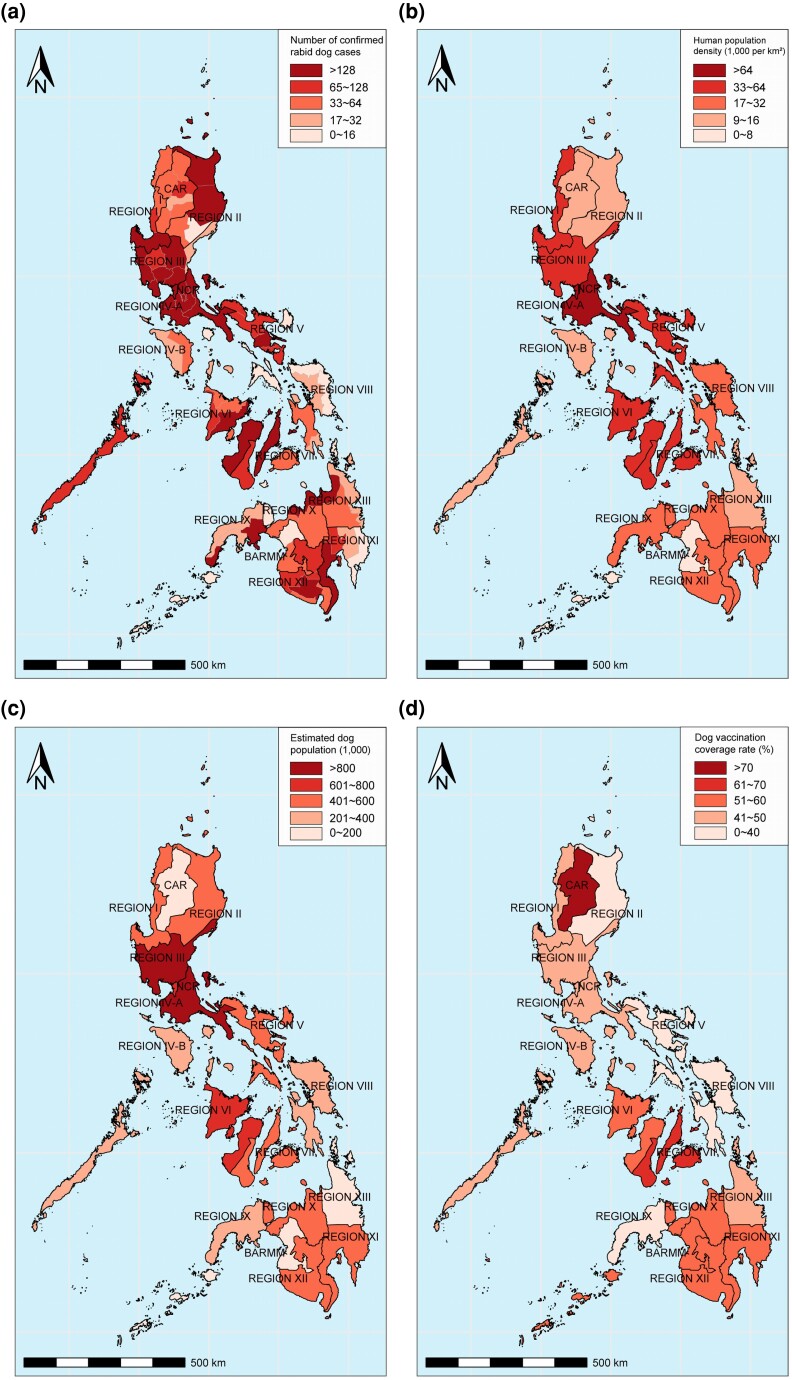
a) Geographic distribution of confirmed dog rabies between 2011 and 2022; b) human population in 2020 retrieved on September 12, 2023 from Philippine Statistic Authority from https://psa.gov.ph/statistics/census/projected-population; c) average dog population and d) vaccination coverage between 2018 and 2019 according to Bureau of Animal Industry statistics (unpublished data).

Brain tissues of 35,700 animals from all 17 administrative regions were collected during rabies surveillance in the Philippines between 2011 and 2022 (Fig. 1a). Of these, 10,225 (27.9%) were confirmed RABV-positive by fluorescent antibody test (FAT), with dogs constituting the vast majority of the positive cases (97.47%, 9,966/10,225), followed by cats (*n* = 201), cattle (*n* = 32), pigs (*n* = 12), goats (*n* = 12), buffalo (*n* = 1), and monkey (*n* = 1) (supplementary table S1, Supplementary Material online). The antirabies immunization records revealed that 94% (*n* = 9,364) of the rabid dogs had no or unknown history of vaccination. Of 7,764 rabid dogs with age information, 61.8% (*n* = 4,797) were under 12 months including 1,763 (22.7%) puppies younger than 3 months. Supplementary table S1, Supplementary Material online also showed that animal rabies incidence largely differed in 17 administrative regions with region III reporting the highest (1,976), while the Bangsamoro Autonomous Region in Muslim Mindanao reporting the lowest number of animal rabies cases (*n* = 10) in the 12-year surveillance period.

### RABV Genome Sequencing and Phylogenetic Analysis

**Fig. 2.**
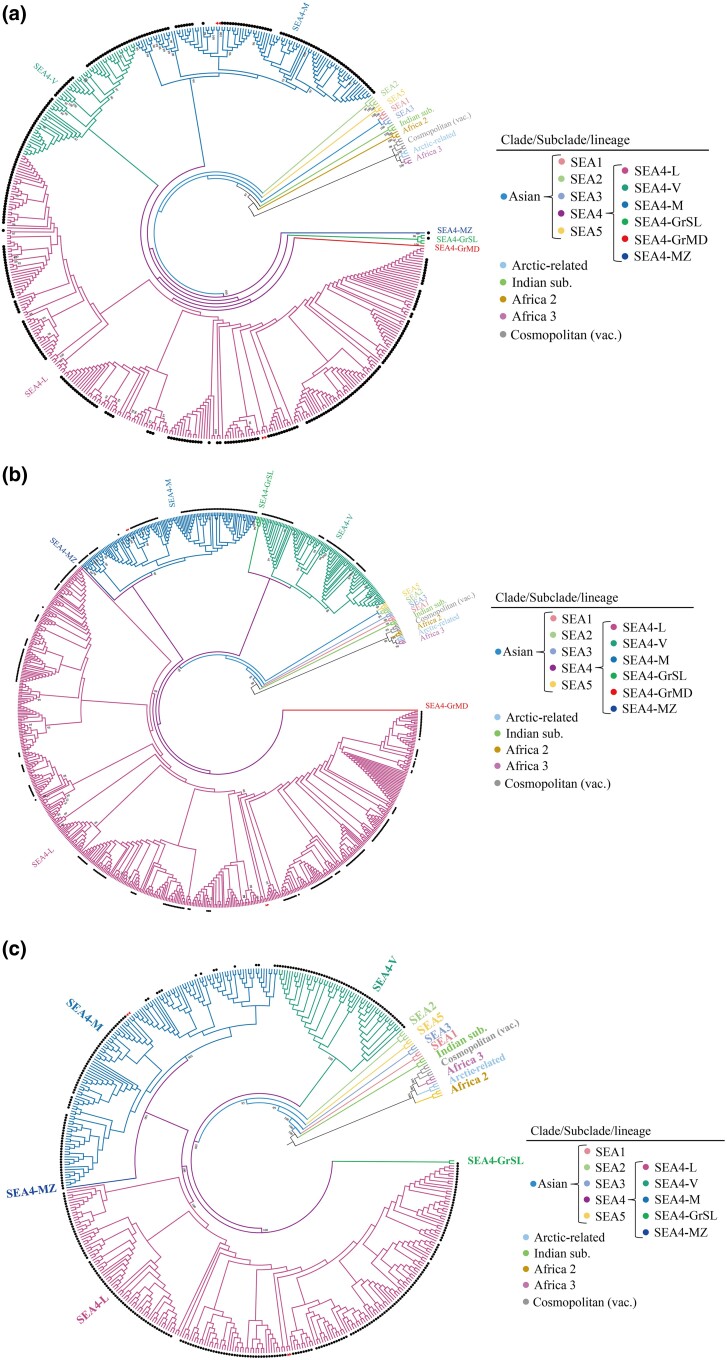
Maximum likelihood phylogeny of RABVs in the Philippines. Bootstrap values = 1,000. a) Phylogenetic analysis of 464 full RABV N sequences showed that all of RABVs in the Philippines belongs to SEA4 subclades and six further lineages. b) Phylogenetic analysis of 772 full RABV G sequences showed the same classification. c) Phylogenetic analysis of 443 RABV complete genome sequences. The dots represent the field strains obtained in this study, and the triangles represent the strains from cross-island transmission.

**Fig. 3.**
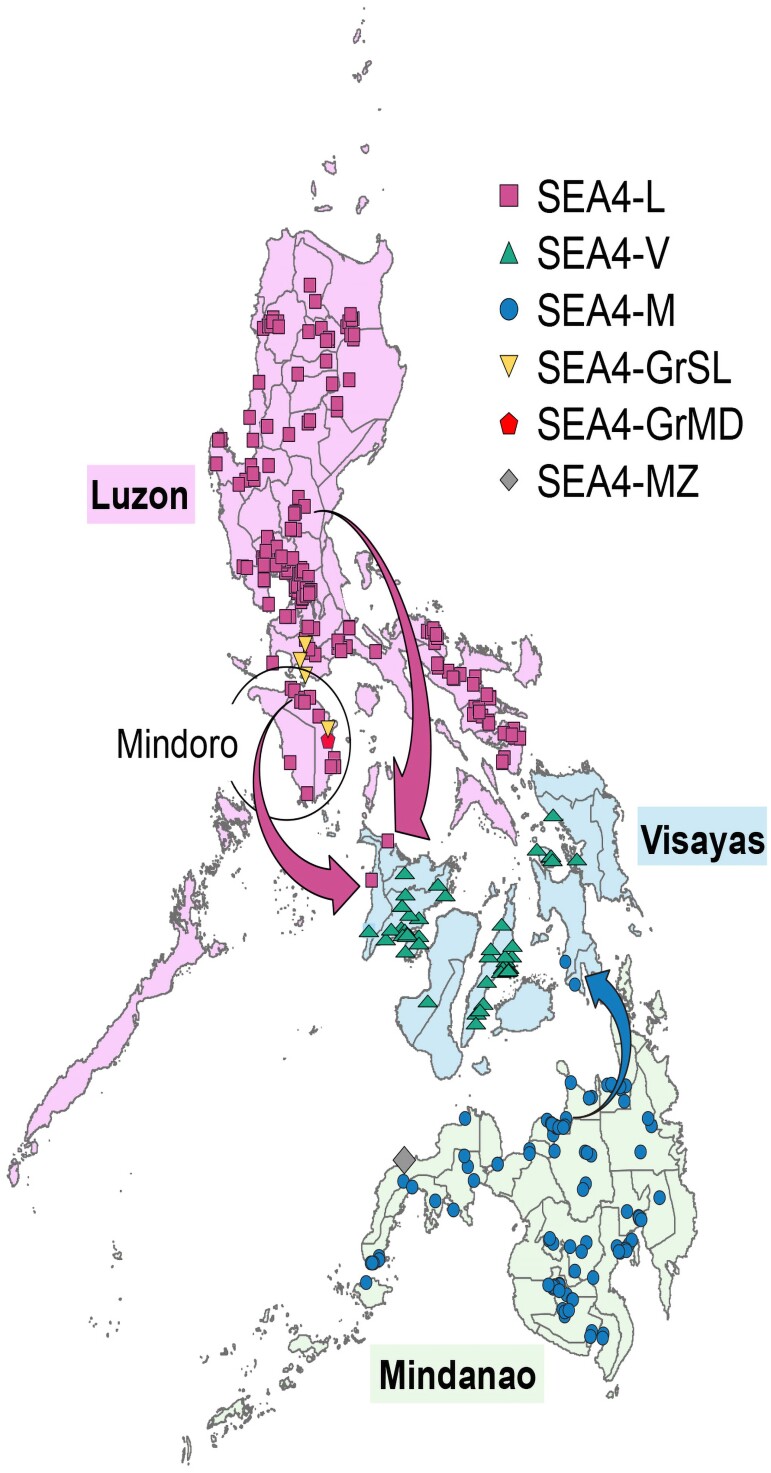
Geo-distribution of six SEA4 lineages in the Philippines. The arrows showed inter-island transmissions of different RABV lineages, respectively, from Luzon and Mindanao to the neighboring areas in Visayas.

Since the start of China/Philippines collaborative project on molecular epidemiology and evolutionary dynamics of RABVs in the Philippines in 2019, a total of 368 positive brain tissue samples representing different outbreaks in different locations between 2018 and 2022 (supplementary fig. S1, Supplementary Material online) were collected and sent by FTA cards to the reference laboratory for rabies in China for RABV genome sequencing (the brain specimens collected before 2018 were not stored, therefore not available for this study). From 368 positive samples shipped by FTA cards, 353 RABV complete genome sequences with a genome coverage of exceeding 99% were obtained through meta-transcriptomics (MTT) by Novogene Co., Ltd (Novogene, Beijing) (supplementary table S2, Supplementary Material online), which were then deposited in the GenBank database with accession numbers OR971323-OR971675 (supplementary table S3, Supplementary Material online). Thirteen cards produced only partial gene sequences and the last two produced nothing. The result demonstrated a robust performance of FTA cards in cross-country shipping of RABV brain samples. In the phylogenetic analysis, the complete N, G genes and complete genome of these 353 isolates, along with other previously published sequences of Philippine RABVs and representative reference sequences from GenBank, were used to determine the complete phylogenetic characteristics of RABVs in the Philippines, respectively. All showed the same results that RABVs circulating in the Philippines were solely classified into the SEA4 subclade within the Asian clade, confirming the presence of a subclade unique to the Philippines (Fig. 2) (Saito et al. 2013; Tohma et al. 2014, 2016). The SEA4 subclade could further be divided into six lineages, SEA4-L, SEA4-V, SEA4-M, SEA4-GrSL, SEA4-GrMD, and SEA4-MZ, of which the former three were predominant in the country and, surprisingly correlated strongly to their geographic distributions in three major islands, i.e. lineage SEA4-L restricted to and circulating mainly in Luzon, while SEA4-V and SEA4-M mainly in Visayas and Mindanao, respectively. The other three were minor lineages circulating only in very small areas (Fig. 3). In the previous study, none of SEA4-L was found in the Mindoro island of Luzon from 2004 to 2010 (Saito et al. 2013). Our surveillance revealed that lineage SEA4-L has emerged in Mindoro since 2019 (Fig. 3, supplementary table S3, Supplementary Material online). Additionally, it further identified the transmission of two SEA4-L strains (R6-2019-1399 and R6-2021-4511) from Luzon to Visayas, and the transmission of two SEA4-M strains (R8-2021-019 and R8-2021-020) from Mindanao to Visayas (Figs. 2 and 3). In addition, the G gene-based phylogenetic analysis (Fig. 2b) also revealed that the two strains (RV/R4B.PHL/2013/Tra-487 and RV/R4B.PHL/2013/Tra-486) from region IV-B in Luzon belong to the SEA4-V lineage, indicating the cross-island transmission. This result indicated that the transmission between two adjacent major islands might occur due to yet-to-be-identified factors, although it is rare. The results also revealed that the earliest RABV sequence (strain 94273PHI, accession number EU086201) identified in the Philippines in 1994 belongs to minor lineage SEA4-GrSL, while SEA4-MZ is a new minor lineage identified from region IX in Mindanao in our study. The phylogenetic tree based on 353 complete genomes together with all 81 Philippine sequences from publications and nine reference sequences from GenBank showed only five subclades, due to the complete genome of the strain in SEA4-GrMD was not available in GenBank (Fig. 2c).

### Temporal Dynamics and Spread of RABVs

**Fig. 4.**
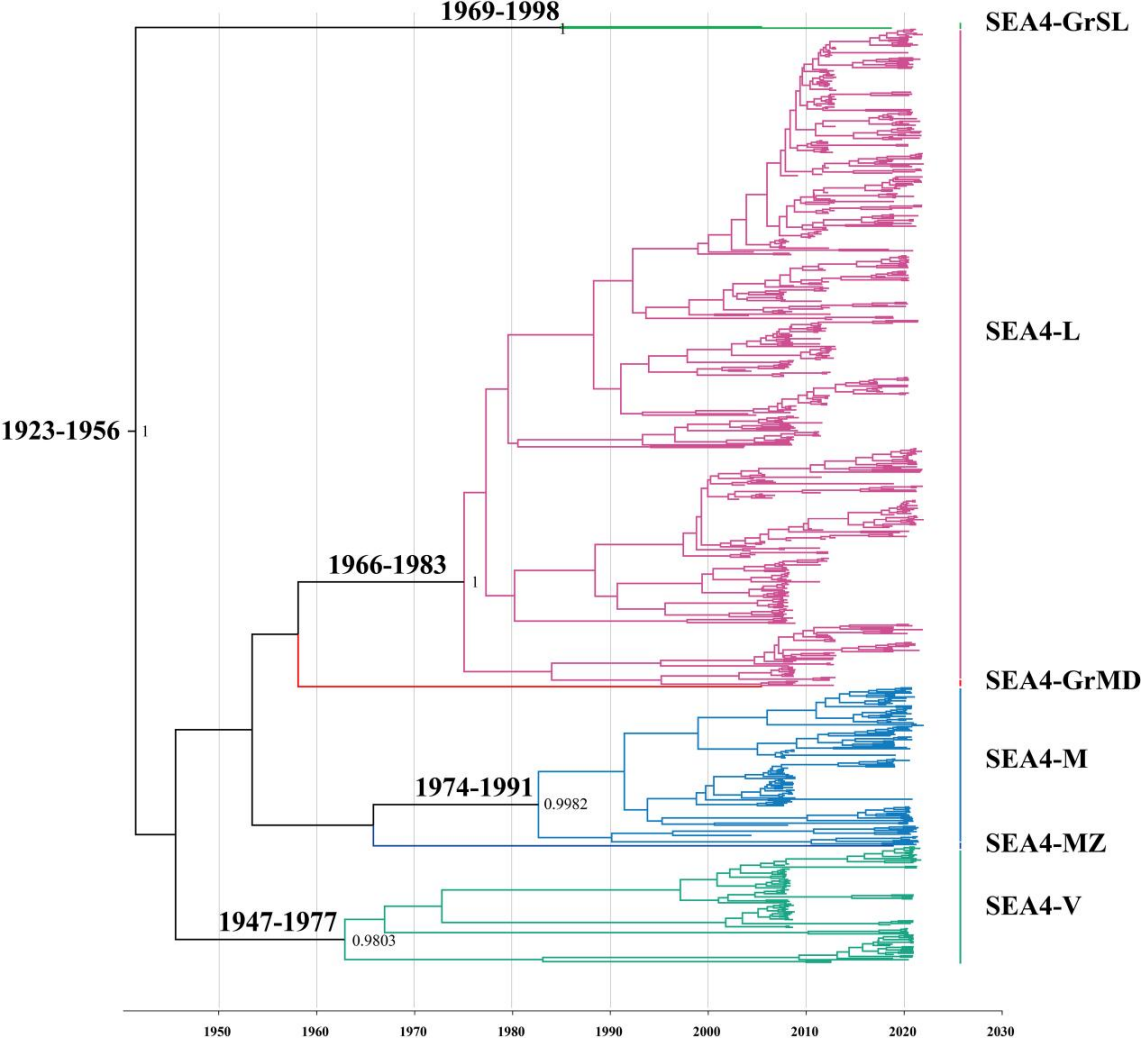
G gene-based maximum clade credibility tree of rabies viruses. The time to most recent common ancestor (TMRCA) of the SEA4 lineages and their 95% highest posterior density values are indicated. The TMRCA of the SEA4-GrMD and SEA4-MZ lineages could not be estimated since each lineage consists of only one strain.

Results of the BEAST evolutionary analysis showed that the mean rate of nucleotide substitution for the tested RABVs was 2.8113 × 10^−4^ substitutions per site per year (95% highest posterior density 2.3796 to 3.1973 × 10^−4^ substitutions per site per year). Our results showed a log marginal likelihood of −19,706.8537 for the Mhet model and −20,346.5302 for the Miso model, resulting in a log Bayes factor (log BF) of 639.68. This value significantly exceeds the threshold of 5, thereby providing the evidence that the dataset encapsulates a robust temporal signal. In addition, this finding is consistent with the previous analysis of the mean rate of evolutionary change performed on global dog-related RABV (Troupin et al. 2016). However, the estimates varied in the following ascending order L–N–G–P–M (Table 1). Differences in evolutionary rates among the clades and subclades were not significant. However, only the M gene had a nucleotide substitution rate considerably higher than those of N and L genes, which is an indicator of weaker selective constraints. The research estimated the time to the most recent common ancestor (TMRCA) of SEA4, which revealed that the lineages in the subclade likely shared a single common ancestor between 1923 and 1956, with an average of 1941 (Fig. 4).

**Table 1:** The pressures under selection of five RABV genes in the Philippines

Dataset	Gene	*d_N_*/*d_S_*	SLAC^a^	FEL^a^	MEME-internal^a^	FUBAR^b^
RABVs (*n* = 353)	N	0.045	…	…	46	…
	P	0.113	…	…	30	…
	M	0.062	…	…	…	…
	G	0.092	…	…	482,485	…
	L	0.039	…	…	…	…

*d_N_*/*d_S_* ratios are calculated using SLAC.

^a^Codons with *P* < 0.05.

^b^Codons with posterior of probability > 0.95.

**Fig. 5.**
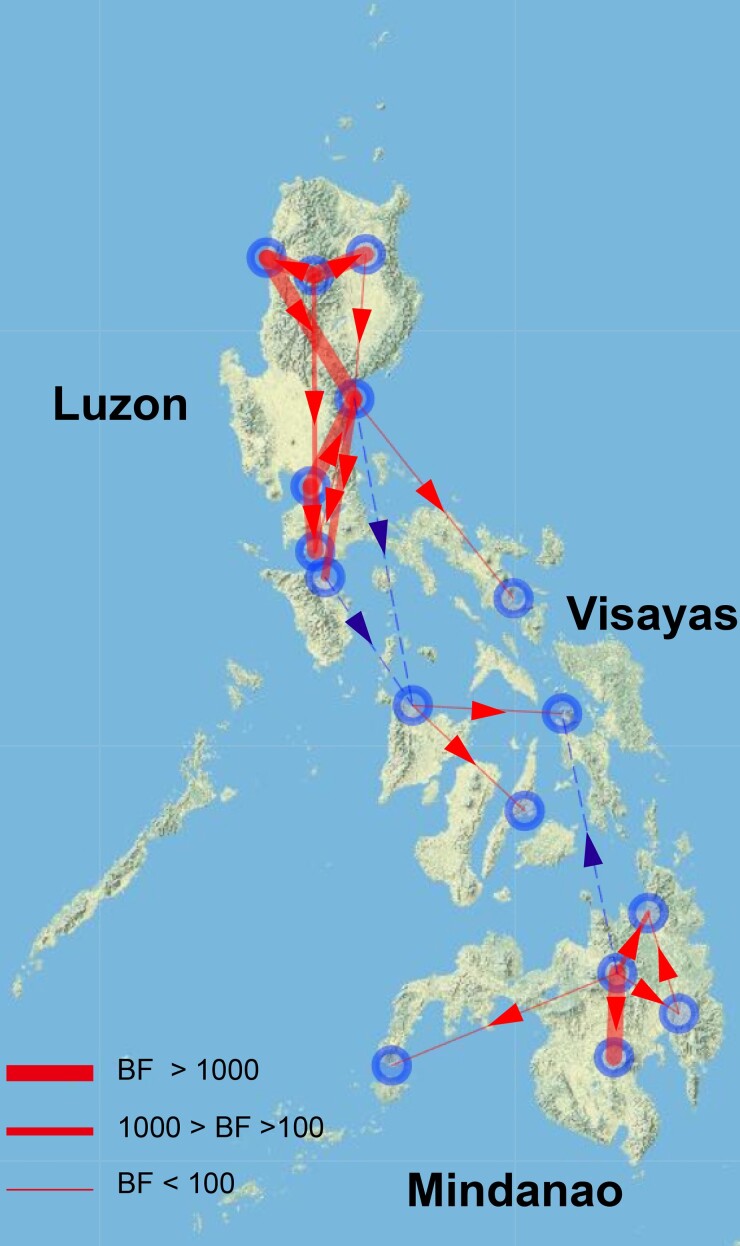
The BSSVS analysis was conducted using BEAST software version 1.10.4 on 724 rabies virus G gene sequences, each annotated with precise geographic and temporal information. This analysis inferred potential transmission routes among the three major islands based on the Bayesian stochastic search variable selection approach. Transmission events between geographic locations were considered supported if the Bayes factor (BF) > 3 and posterior probability (PP) > 0.5. The red lines indicate migration events with varying levels of Bayesian support, where the line width reflects the BF values, ranging from 3.36 to 59,819.85. Thicker lines correspond to higher BF values, indicating greater confidence in the inferred transmission route. Solid lines depict intra-island transmission, while dashed lines denote inter-island transmission.

Further analysis of significant translocation pathways of RABV subclades by BEAST based on the G gene revealed that rabies endemic in the Philippines is mainly dominated by intra-island (Bayes factor 59,819.84777) rather than inter-island transmission, indicating that ocean barrier plays important roles in shaping rabies transmission in the country. The inter-island rabies transmission among these three major island regions is rare, only a few Luzon-to-Visayas or Mindanao-to-Visayas transmissions were identified (Fig. 5, supplementary table S4, Supplementary Material online).

### Variation in Evolutionary Rates and Selection Pressures Along the RABV Genome

Results of the Bayesian skyline model analysis showed that the mean rate of nucleotide substitution for the five concatenated genes was 2.8113 × 10^−4^ substitutions per site per year (95% highest posterior density 2.3796 to 3.1973 × 10^−4^ substitutions per site per year) (supplementary fig. S2, Supplementary Material online). This finding is consistent with the previous analyses of evolutionary change performed on dog-related RABVs (Troupin et al. 2016). To determine if the variation in rates of evolutionary change might result from differing selection pressures, we first compared the ratio of nonsynonymous (*d_N_*) to synonymous (*d_S_*) substitutions per site. This analysis was performed on each of the five RABV genes of the Philippine RABV. For each gene, the *d_N_*/*d_S_* ratios are very similarly low and follow the ascending order of L–N–M–G–P genes (Table 1). Furthermore, we explored the number of positively selected sites using several different approaches (Single Likelihood Ancestor Counting [SLAC], Fast Unbiased Bayesian Approximation [FUBAR], Mixed Effects Model of Evolution [MEME], and Fixed Effects Likelihood [FEL]) (Kosakovsky Pond and Frost 2005; Murrell et al. 2012, 2013). Amino acid sites under pervasive positive selection by MEME include one amino acid site for the N gene (46), one for the P gene (30), and two for the G gene (482, 485). Together, these results are generally indicative of strong purifying selection among all sites and branches of the RABV phylogeny, which is consistent with previous study on complete genome sequence analysis of global canine rabies viruses (Troupin et al. 2016).

### The Risk Assessment Model for RABV Transmission

According to the result of the multivariate generalized linear mixed model (GLMM), four independent variables showed a positive correlation with rabies incidence in the Philippines (supplementary table S5, Supplementary Material online). Mean temperature in the wettest quarter (OR: 1.164, 1.023 to 1.324) was significantly correlated with the positive rate indicating that the high temperature and humidity environment was conducive to rabies incidence in dogs, which was consistent with the previous study (Lachica et al. 2020). The Chiroptera richness (OR: 1.065 [1.028, 1.102]) has been found to be associated with an increase in the positive rate of RABV, whereas the Rodentia and Carnivora richness did not show a statistically significant impact. The optimal travel time to healthcare by motorized transportation (Motorized to Healthcare institution, OR: 1.009, 1.004 to 1.013) and percentage of agriculture (OR: 1.014, 1.005 to 1.023) were also positively correlated with dog rabies incidence, suggesting that the risk of RABV transmission in dog population may increase in areas where a variety of crops are planted and medical/health conditions are poor.

## Discussion

Swift sequencing and analysis can inform public health responses and containment strategies (Jaswant et al. 2024). However, genomic research capacity is often constrained by limited laboratory infrastructure, costs, supply chain challenges, and other issues (Brunker et al. 2020). Our study established and optimized the procedure using FTA cards for convenient and safe preservation and long-distance transportation of rabies brain tissue samples for highly effective detection and sequencing of RABV. Using this method, 368 positive brain tissues were shipped from the Philippines to the reference laboratory in China in under a week, and 366 of them produced successful detection of RABV genes by high-throughput sequencing with 353 full genomes obtained. The result demonstrated the high reliability of FTA cards for storage, transport, and molecular detection of rabies samples, highlighting their utility in facilitating international collaboration within the field of routine sequencing.

Our study using a large number of sequences obtained in recent years from continuous surveillance across the entire country, along with the representative sequences published in GenBank during the past decade, has provided the most comprehensive update on the genetic diversity and transmission dynamics of RABV in the Philippines. It has been confirmed that RABVs circulating in the Philippines belong only to the SEA4 subclade, which is the country's unique subclade since it has not been reported in any other countries. Previous studies have shown that the spread of rabies is influenced by geographical boundaries, with the ocean being a significant barrier (Saito et al. 2013; Tohma et al. 2014). The Philippines is a country composed of multiple islands, primarily divided into three major islands: Luzon, Visayas, and Mindanao. Our results showed that this topography shaped a specific rabies transmission in the Philippines, making the RABVs evolve into three major lineages, respectively, corresponding to the three geographical regions. Rabies endemic in each island is dominated by a lineage, and inter-island transmission among them was rare. This finding highlights the critical role of the ocean and other bodies of water in the Philippines as natural barriers that restrict rabies transmission. It suggests that independently implementing dog-mediated rabies elimination efforts in major island regions, in stages, is both feasible and potentially cost-effective. This approach may be a practical alternative when nationwide elimination is challenging due to limited socioeconomic resources. Of the three minor lineages, SEA4-GrSL was first reported in 2013 (Saito et al. 2013), and detected again in the present study from a sample (R4A-2019-772) collected in 2019 from Batangas province of Luzon IV-A region, indicating while the lineage's spread is limited, it continues to contribute to local transmission cycles within the region, despite not being widely distributed across larger geographical areas. However, the SEA4-GrMD lineage previously circulating in region IV was not detected in our study, but a new minor lineage, SEA4-MZ, was first identified in the sample R9-2019-311 collected in 2019 from the Zamboanga Del Norte province of the IX region of Mindanao island. This result underscores the dynamic nature of viral evolution and highlights the need for continuous surveillance and updating of genetic databases to accurately track emerging variants. Although rarely happening, a few inter-island transmissions were observed between regions IV and VI as well as VIII and X, resulting in the spread of RABV lineages from Luzon and Mindanao to nearby areas in Visayas. These inter-island transmission events were due to human-mediated dog movement, which was supported by the nonlocal diffusive model that the distribution of infected dogs from 2011 to 2021 closely mirrors the distribution of flights or ships across the Philippines (unpublished data). In the Philippines, it is a common practice for people to travel with their dogs, including cross-island travels by boat or plane. The frequent ferry transports with dogs pose a significant risk for RABV spread from infected areas to a rabies-free zone since vaccination certificates and other necessary documents are not checked thoroughly at small ports. The 2007 Anti-rabies Act covers regulations on responsible pet ownership, pet vaccination, surveillance, and quarantine (Tohma et al. 2016). With insufficient and inadequate enforcement of the act, as well as limited financial and technical resources, monitoring all traveling dogs is difficult. The import and transportation of potentially infected dogs without inspection may increase the risk of rabies introduction to new areas (Tohma et al. 2016).

The World Health Organization (WHO), World Organization for Animal Health (WOAH) and the Food and Agriculture Organization of the United Nations (FAO) have set a target for global elimination of dog-mediated human rabies by 2030, but mass dog vaccination programs remain still limited in Africa and Asia. Our results showed that 94% of rabid dogs were unvaccinated in the Philippines. The low vaccination coverage among adult dogs and the absence of maternal antibodies in puppies led to high rabies incidence in dogs younger than 12 months (61.8%). The immunization policy for dogs in the Philippines is to provide prime vaccination at three months old. However, since the vaccination of puppies is not diligently practiced, this has resulted in a high incidence of rabies, particularly in puppies less than 6 months of age, as the data show that 22.7% (1,763/7,764) of dog rabies cases in our study were under 3 months old (supplementary table S1, Supplementary Material online). Therefore, as per the recommendation of the WHO, it is important to strictly adhere to the standard vaccination procedure, particularly for puppies under 3 months of age in vaccination campaigns in rabies-endemic regions (WHO, 2018). It is also notable that the majority of rabid dogs (7,726/9,966) were owned pets (supplementary table S1, Supplementary Material online), indicating a low awareness of dog vaccination. In addition, high temperature and humidity climate, and poor medical and health conditions in rural areas, also increased the risk of rabies transmission by our analysis. These factors may contribute to the ease of virus spread among dog populations, thereby increasing the risk of human infection with rabies.

In summary, with the comprehensive dataset, our study found the evidence of very strong phylogeographical structure with little inter-island transmission, suggesting that the nation can develop and implement island-independent rabies control measures to eliminate dog-mediated rabies gradually from one island to another in case of facing socioeconomic challenges. For example, the stray dog impounding program in Davao City showed a significant reduction in reported rabies cases in the city (Lachica et al. 2019). In addition, the risk of importation/incursions of rabies into other areas is still there in the Philippines, although apparently rare. Thus, application with nationwide genomic data in the surveillance of rabies is necessary and should be extended in future, which will be helpful for identifying the orphan branches or clades circulated in this country and understanding the genetic diversity of RABV within the Philippines.

## Materials and Methods

### Sample Collection, Detection, and Transportation

The Bureau of Animal Industry, through its Veterinary Laboratory Division-Animal Disease Diagnosis and Reference Laboratory (VLD-ADDRL) and the 15 Regional Animal Disease Diagnostic Laboratory in the Philippines, conducted the nationwide animal rabies surveillance program, which specifically targeted dogs exhibiting abnormal behaviors, clinical signs consistent with rabies, and a history of biting humans or other domestic animals. Brain tissues from these suspect rabid dogs and other animals were collected and examined using WOAH protocol of the direct FAT with FITC-conjugated antirabies monoclonal antibody (Fujirebio Diagnostics Inc., 800-092, Japan) (Rupprecht et al. 2018). The epidemiology information obtained during the period 2011 to 2022 formed the basis for this research. In total, 368 RABV-positive brain tissues from different areas and different dates from 2018 to 2022 were obtained and their 40% homogenates were loaded onto FTA cards (Whatman, WB120205, England) (Picard-Meyer et al. 2007; Sakai et al. 2015). After air-drying at room temperature for 2 h, FTA cards were sealed in a self-sealing plastic bag and sent to the WOAH Reference Laboratory for Rabies in China for RABV genome sequencing.

### High-Throughput Sequencing of RABV Genome Sequences

The circle patches containing inactivated brain tissue homogenates in FTA cards were cut and soaked using 500 μL TE buffer and were centrifuged at 12,000 r/min at 4 °C for 5 min to recover the supernatant. Then, 200 μL supernatant was lysed by TRIzol reagent (Invitrogen, 15596-026, America), and subsequently sequenced using MTT on the Illumina HiSeq 2500 platform with paired-end reads of 150 bp (PE150). After removal of the host genomic and RNA sequences, coverage and depth of viral genomes were calculated with SAMtools (v1.9) (Li et al. 2009) based on the SAM files from Bowtie 2 (v2.4.4) (Langmead and Salzberg 2012). The remaining reads were mapped to the protein Preference Viral Database for viral discovery (Bigot et al. 2019) and then de novo assembled using Diamond v2.1.8 and MEGAHIT v2.1.9 to obtain whole genome sequences of RABV.

All RABV sequences were manually inspected for accuracy, such as the presence of intact open reading frames, using BioEdit (http://www.bioedit.com/). The alignment of these newly sequenced genomes, along with complete genome sequences of other Philippine RABV strains downloaded from GenBank, was constructed using ClustalW2 with default parameters (http://www.ebi.ac.uk/Tools/msa/clustalw2/) and manually adjusted when necessary.

### Phylogenetic Analysis

Phylogenetic analyses of 464 full RABV N sequences (353 from this study, 81 published and 30 reference sequences from GenBank), 772 full RABV G sequences (353 from this study, 389 published and 30 reference sequences from GenBank), as well as 443 RABV complete genome sequences (353 from this study, 81 published and nine reference sequences from GenBank) were performed using the maximum likelihood of MEGA X software with the T92 + G + I substitution model selected using ModelFinder and estimated using 1,000 bootstrap replicates. Branches with <70% bootstrap support were collapsed using the iTOL for phylogenetic trees.

### Estimates of RABV Evolutionary Dynamics and Time-scale

To investigate the linear evolutionary rates of five RABV genes, we used the Bayesian Markov chain Monte Carlo in BEAST version 1.8.2 package (Drummond and Rambaut 2007) by incorporating information on the sampling date (day-month-year) of the Philippine RABVs (the samples without sampling date were excluded). For these analyses, we selected the general time reversible model as the substitution model and gamma plus invariable sites as the site heterogeneity model based on the calculations of the Model Generator (Keane et al. 2006). An uncorrelated log normal relaxed molecular clock model and the constant size model as a coalescent tree prior were also selected for the analyses, which were run for 300 million steps with sampling at every 30,000 states (Drummond et al. 2006). The BEAGLE parallel computation library was used to enhance the speed of the likelihood calculations (Ayres et al. 2012). Finally, the resulting log file was checked using TRACER version 1.5 (http://tree.bio.ed.ac.uk/software/tracer) to confirm that all effective sample sizes were >200. Based on the analyses, estimations were made of the rates of nucleotide substitution for each RABV gene.

To investigate the phylogeographic spread of RABVs in the Philippines, the complete G genes in the country were used to predict transmission routes of RABV lineages with a symmetric Bayesian stochastic search variable selection (BSSVS) approach, in which we applied a Bayes factor to determine the best-supported transmission event between two geographic locations. Bayes factors were calculated by SpreaD3 software with a value >3 as cutoff (Lemey et al. 2009; Bielejec et al. 2016).

### Selection Pressures Analysis

The Datamonkey server (http://www.datamonkey.org) was used to reveal the selection pressures acting on each RABV gene (Pond and Frost 2005; Weaver et al. 2018). We compared the numbers of nonsynonymous (*d_N_*) and synonymous (*d_S_*) substitutions per site for the five RABV genes and phylogenetic lineages using the SLAC, FEL, MEME, and FUBAR models (Kosakovsky Pond and Frost 2005; Murrell et al. 2012; Murrell et al. 2013). Only codon positions with a *P* < 0.05 for the SLAC, FEL, and MEME models and with a posterior of probability > 0.95 for the FUBAR method were considered as containing evidence for positive selection.

### Risk Assessment Model of RABV

A generalized linear mixed (GLM) model was employed to evaluate the impact of 33 ecological and social factors (supplementary table S6, Supplementary Material online) on RABV prevalence with the epidemiological data obtained between 2011 and 2022, utilizing the glmmTMB function from the R package glmmTMB. All raster-type map layers of these ecological and social factors were overlapped onto the regional-level vector digital map of the Philippines. These factors were calculated for the average and summation by using the zonal statistical calculation technique. The processing and analysis of the raster data were conducted by using ArcGIS 10.7 (Environmental Systems Research Institute Inc., Redlands, CA, USA). The average annual positive rate of rabid dogs at regional-level from 2011 to 2022 was designated as response variable for this model. Additionally, the 33 variables processed to the regional-level were incorporated into the model as explanatory variables. Initially, a univariate model was applied to identify potential contributing factors, retaining only variables with *P*-values less than 0.25 for inclusion in the subsequent multivariate model. To address multicollinearity within the GLM model, the variance inflation factor was calculated using the “vifstep” function from the R package “usdm”. Variable selection was performed using a backward elimination procedure based on the Akaike information criterion (AIC), where the variable whose removal resulted in the greatest AIC reduction was excluded at each step. The final optimal model only included variables with *P*-values less than 0.05. All statistical analyses were conducted using R software (version 4.2.1, R Development Core Team, 2020).

## Supplementary Material

msaf007_Supplementary_Data

## Data Availability

Data supporting the findings of this work are available within the paper and its supplementary information files, Supplementary Material online. All sequences generated as part of this study are deposited in GenBank (accession codes: OR971323-OR971675). Source data are provided in this paper.
