# Supplementary material for: Phylogeography Analysis Reveals Rabies Epidemiology, Evolution, and Transmission in the Philippines
Source: Mol Biol Evol. 2025 Feb 12;42(2):msaf007. doi: 10.1093/molbev/msaf007 (PMC11815495; doi:10.1093/molbev/msaf007)
Supplement: msaf007_Supplementary_Data [file msaf007_supplementary_data.zip › Supplementary Table 1.pdf]

Supplementary table S1. Sample information of animal rabies surveillance between 2011 to 2022 in the Philippines.

| Islands | Region | Number of surveillance sample | Numbers of RABV-postive | Number of rabid animal species | | | | | | | Age (mos.) of rabid dogs | | | | | Ownership of rabid dogs | | | | Vaccination of rabid dogs | | | |
|---|---|---|---|---|---|---|---|---|---|---|---|---|---|---|---|---|---|---|---|---|---|---|---|
| | | | | Dog | Cat | Cattle | Pig | Goat | Buffalo | Monkey | ≤3 mos | ≤12 mos. | > 12 mos. | Unknown | Total | Pet | ownerless | Unknown | Total | Yes | No | Unknown | Total |
| Luzon | CAR | 1182 | 270 | 262 | 6 | 1 | 0 | 1 | 0 | 0 | 51 | 67 | 57 | 87 | 262 | 216 | 41 | 5 | 262 | 16 | 136 | 110 | 262 |
| | NCR | 3104 | 878 | 841 | 36 | 1 | 0 | 0 | 0 | 0 | 131 | 207 | 281 | 222 | 841 | 646 | 178 | 17 | 841 | 33 | 178 | 630 | 841 |
| | I | 3565 | 853 | 836 | 12 | 4 | 0 | 1 | 0 | 0 | 208 | 263 | 256 | 109 | 836 | 737 | 95 | 4 | 836 | 50 | 661 | 125 | 836 |
| | II | 801 | 395 | 379 | 15 | 0 | 0 | 0 | 0 | 1 | 70 | 112 | 92 | 105 | 379 | 326 | 46 | 7 | 379 | 21 | 152 | 206 | 379 |
| | III | 8343 | 1976 | 1920 | 46 | 8 | 1 | 1 | 0 | 0 | 322 | 566 | 593 | 439 | 1920 | 1533 | 375 | 12 | 1920 | 133 | 953 | 834 | 1920 |
| | IV-A | 2984 | 1039 | 1026 | 11 | 0 | 2 | 0 | 0 | 0 | 154 | 301 | 384 | 187 | 1026 | 764 | 215 | 47 | 1026 | 52 | 214 | 760 | 1026 |
| | IV-B | 632 | 169 | 162 | 6 | 1 | 0 | 0 | 0 | 0 | 37 | 46 | 52 | 27 | 162 | 140 | 17 | 5 | 162 | 9 | 44 | 109 | 162 |
| | V | 1960 | 554 | 541 | 9 | 4 | 0 | 0 | 0 | 0 | 63 | 272 | 140 | 66 | 541 | 375 | 165 | 1 | 541 | 47 | 434 | 60 | 541 |
| Visayas | VI | 2252 | 1041 | 1024 | 13 | 3 | 0 | 1 | 0 | 0 | 162 | 339 | 273 | 250 | 1024 | 778 | 230 | 16 | 1024 | 72 | 437 | 515 | 1024 |
| | VII | 3897 | 778 | 762 | 13 | 1 | 0 | 2 | 0 | 0 | 101 | 184 | 235 | 242 | 762 | 477 | 283 | 2 | 762 | 31 | 464 | 267 | 762 |
| | VIII | 788 | 140 | 139 | 1 | 0 | 0 | 0 | 0 | 0 | 25 | 46 | 51 | 17 | 139 | 91 | 46 | 2 | 139 | 9 | 72 | 58 | 139 |
| Mindanao | IX | 493 | 298 | 283 | 14 | 0 | 1 | 0 | 0 | 0 | 61 | 85 | 65 | 72 | 283 | 202 | 77 | 4 | 283 | 18 | 128 | 137 | 283 |
| | X | 1636 | 588 | 572 | 4 | 7 | 1 | 4 | 0 | 0 | 130 | 164 | 153 | 125 | 572 | 456 | 113 | 3 | 572 | 45 | 340 | 187 | 572 |
| | XI | 1207 | 370 | 361 | 7 | 2 | 0 | 0 | 0 | 0 | 73 | 107 | 107 | 74 | 361 | 293 | 66 | 2 | 361 | 25 | 246 | 90 | 361 |
| | XII | 1683 | 601 | 593 | 6 | 0 | 1 | 1 | 0 | 0 | 132 | 190 | 163 | 108 | 593 | 474 | 116 | 3 | 593 | 28 | 237 | 328 | 593 |
| | XIII | 1158 | 265 | 256 | 1 | 0 | 6 | 1 | 1 | 0 | 42 | 82 | 63 | 69 | 256 | 211 | 45 | 0 | 256 | 13 | 113 | 130 | 256 |
| | BARMM | 15 | 10 | 9 | 1 | 0 | 0 | 0 | 0 | 0 | 1 | 3 | 2 | 3 | 9 | 7 | 2 | 0 | 9 | 0 | 7 | 2 | 9 |
| Total | | 35,700 | 10,225 | 9,966 | 201 | 32 | 12 | 12 | 1 | 1 | 1763 | 3034 | 2967 | 2202 | 9966 | 7726 | 2110 | 130 | 9966 | 602 | 4816 | 4548 | 9966 |
