# Supplementary material for: Phylogeography Analysis Reveals Rabies Epidemiology, Evolution, and Transmission in the Philippines
Source: Mol Biol Evol. 2025 Feb 12;42(2):msaf007. doi: 10.1093/molbev/msaf007 (PMC11815495; doi:10.1093/molbev/msaf007)
Supplement: msaf007_Supplementary_Data [file msaf007_supplementary_data.zip › Supplementary Table 2.pdf]

Supplementary table S2. Sequencing coverage and assembly statistics (Reference strain CVS-11, GenBank: GQ918139.1)

Sample_id	Genome length	Mapped reads	Mean depths	Genome coverage	Note
CAR-2019-085	11925	1085065	27297.23	99.98%	
CAR-2019-092	11927	3921154	98628.84	100.00%	
CAR-2019-098	11924	1253456	31536.13	99.97%	
CAR-2019-099	11926	28518	717.37	99.99%	
CAR-2019-103	11925	1360943	34237.56	99.98%	
CAR-2019-111	11926	1258151	31648.94	99.99%	
CAR-2019-208	11922	305642	7691.04	99.96%	
CAR-2021-015	11926	2184480	54950.86	99.99%	
CAR-2021-016	11926	870487	21897.21	99.99%	
CAR-2021-022	11924	1045244	26297.65	99.97%	
CAR-2021-032	11924	680289	17115.62	99.97%	
CAR-2021-033	11926	691003	17382.27	99.99%	
CAR-2021-034	11922	737843	18566.76	99.96%	
CAR-2021-040	11926	71249	1792.28	99.99%	
CAR-2021-044	11926	626159	15751.11	99.99%	
CAR-2021-046	11922	262498	6605.39	99.96%	
CAR-2021-074	11926	690405	17367.22	99.99%	
CAR-2021-075	11926	182623	4593.90	99.99%	
CAR-2021-092	6992	78	3.35	58.62%	failure
CAR-2022-001	11926	230956	5809.73	99.99%	
CAR-2022-002	11924	41312	1039.38	99.97%	
CAR-2022-004	11926	156536	3937.68	99.99%	
CAR-2022-007	11926	75748	1905.45	99.99%	
CAR-2022-018	11924	126176	3174.51	99.97%	
CAR-2022-020	11926	313173	7877.91	99.99%	
NCR-2019-6154	11924	522993	13158.16	99.97%	
NCR-2019-6544	11922	698358	17573.18	99.96%	
NCR-2019-6661	11922	233183	5867.72	99.96%	
NCR-2020-1676	11924	143216	3603.22	99.97%	
NCR-2020-2509	11924	1374668	34585.74	99.97%	
NCR-2020-2625	11924	2003411	50404.50	99.97%	
NCR-2020-2865	11926	420828	10585.98	99.99%	
NCR-2020-3068	11926	936019	23545.67	99.99%	
NCR-2020-3257	11927	1207628	30375.48	100.00%	
NCR-2020-3323	11927	592115	14893.48	100.00%	
NCR-2021-3600	11927	707490	17795.51	100.00%	
NCR-2021-3654	11927	579726	14581.86	100.00%	
NCR-2021-3728	5958	242	12.19	49.95%	failure
NCR-2021-3901	11927	427833	10761.29	100.00%	
NCR-2021-4011	11927	597623	15032.02	100.00%	

NCR-2022-1945	11926	1152816	28999.23	99.99%	
NCR-2022-1961	11926	730825	18383.99	99.99%	
NCR-2022-2199	11926	674534	16967.99	99.99%	
NCR-2022-2209	11927	887705	22328.46	100.00%	
NCR-2022-2610	11927	575736	14481.50	100.00%	
NCR-2022-2837	11927	694755	17475.18	100.00%	
NCR-2022-2918	11927	181556	4566.68	100.00%	
NCR-2022-2923	11927	488649	12291.00	100.00%	
NCR-2022-2994	11927	924127	23244.58	100.00%	
NCR-2022-2995	11927	1426229	35873.96	100.00%	
NCR-2022-3041	11928	164620	4140.34	100.00%	
NCR-2022-3089	11927	1200461	30195.21	100.00%	
NCR-2022-3192	11927	1797206	45205.15	100.00%	
NCR-2022-3197	11927	551769	13878.65	100.00%	
NCR-2022-3207	11927	292061	7346.21	100.00%	
NCR-2022-3281	11927	2790002	70176.96	100.00%	
NCR-2022-3357	11927	531204	13361.38	100.00%	
NCR-2022-3487	11927	459492	11557.61	100.00%	
NCR-2022-3580	11927	7831206	196978.44	100.00%	
NCR-2022-3616	11927	62078	1561.45	100.00%	
R1-2019-178	11926	268265	6748.24	99.99%	
R1-2019-193	11924	319278	8032.82	99.97%	
R1-2019-196	11924	757507	19058.38	99.97%	
R1-2019-204	11924	441814	11115.75	99.97%	
R1-2019-212	11926	582675	14657.26	99.99%	
R1-2021-161	11924	561943	14138.12	99.97%	
R1-2021-162	11924	306162	7702.83	99.97%	
R1-2021-165	11926	539400	13568.67	99.99%	
R1-2021-166	11922	1890240	47565.17	99.96%	
R1-2022-034	11927	82688	2079.85	100.00%	
R1-2022-040	11927	621880	15642.16	100.00%	
R1-2022-042	11927	1785515	44911.08	100.00%	
R2-2019-7027	11924	200677	5048.90	99.97%	
R2-2019-7465	11924	235111	5915.24	99.97%	
R2-2019-7838	11924	236071	5939.39	99.97%	
R2-2019-8088	11924	73611	1852.00	99.97%	
R2-2019-8166	11924	53499	1346.00	99.97%	
R2-2019-8204	11924	125275	3151.84	99.97%	
R2-2019-8228	11924	195863	4927.78	99.97%	
R2-2021-082	11923	1428076	35932.47	99.97%	
R2-2021-083	11927	149019	3748.28	100.00%	
R2-2021-084	11923	229655	5778.45	99.97%	
R2-2021-087	11927	228	5.73	100.00%	
R2-2021-094	7665	234	9.16	64.27%	failure

R2-2021-106	11923	28461	716.12	99.97%
R2-2021-121	11927	1043	26.23	100.00%
R2-2021-124	11924	228736	5754.85	99.97%
R2-2021-125	11924	364681	9175.13	99.97%
R3-2019-5590	11927	537477	13519.17	100.00%
R3-2019-5829	11927	1261343	31726.58	100.00%
R3-2019-5868	11927	1956826	49220.07	100.00%
R3-2019-6317	11927	234797	5905.85	100.00%
R3-2021-0274	11922	213864	5381.58	99.96%
R3-2021-0282	11922	137729	3465.75	99.96%
R3-2021-0286	11922	395456	9951.08	99.96%
R3-2021-0289	11922	247044	6216.51	99.96%
R3-2021-0294	11922	355980	8957.73	99.96%
R3-2021-0296	11922	64671	1627.35	99.96%
R3-2021-0300	11922	679800	17106.19	99.96%
R3-2021-0304	11927	609141	15321.73	100.00%
R3-2021-0322	11927	761104	19144.06	100.00%
R3-2021-0467	11927	1031641	25948.88	100.00%
R3-2021-0468	11927	191482	4816.35	100.00%
R3-2021-0469	11927	494016	12425.99	100.00%
R3-2021-0472	11927	411346	10346.59	100.00%
R3-2021-0483	11927	608379	15302.57	100.00%
R3-2021-0484	11927	1288168	32401.31	100.00%
R3-2021-0487	11927	433089	10893.49	100.00%
R3-2021-0495	11928	91375	2298.16	100.00%
R3-2021-0498	11927	588170	14794.25	100.00%
R3-2021-0501	11927	366429	9216.79	100.00%
R3-2021-0502	11927	1428802	35938.68	100.00%
R3-2021-0504	11927	204888	5153.55	100.00%
R3-2021-0513	11927	3182088	80039.10	100.00%
R3-2021-0514	11927	1565693	39381.90	100.00%
R3-2021-0543	11927	835395	21012.70	100.00%
R3-2021-0731	11927	647830	16294.88	100.00%
R3-2021-0753	11927	2187900	55032.28	100.00%
R3-2021-3790	11927	619383	15579.35	100.00%
R3-2022-0002	11927	1889358	47523.05	100.00%
R3-2022-0004	11927	1008814	25374.71	100.00%
R3-2022-0006	11927	418119	10516.95	100.00%
R3-2022-0013	11927	1376491	34622.90	100.00%
R3-2022-0014	11927	520556	13093.55	100.00%
R3-2022-0016	11927	1526827	38404.30	100.00%
R3-2022-0017	11927	1440292	36227.69	100.00%
R3-2022-0019	11927	1248478	31402.98	100.00%
R4A-2019-472	11926	814442	20487.39	99.99%

R4A-2019-772	11927	119517	3006.21	100.00%
R4A-2019-857	11926	469670	11814.61	99.99%
R4A-2019-892	11924	321531	8089.51	99.97%
R4A-2019-949	11922	796402	20040.31	99.96%
R4A-2019-989	11922	791594	19919.33	99.96%
R4A-2019-1023	11926	526940	13255.24	99.99%
R4A-2019-1033	11924	293867	7393.50	99.97%
R4A-2019-5622	11922	695151	17492.48	99.96%
R4A-2019-5970	11922	586350	14754.66	99.96%
R4A-2020-0084	11922	220234	5541.87	99.96%
R4A-2020-2377	11924	217639	5475.65	99.97%
R4A-2020-2577	11928	270378	6800.25	100.00%
R4A-2021-1574	11927	1057104	26589.35	100.00%
R4A-2021-1668	11927	983664	24742.11	100.00%
R4A-2021-2633	11927	245136	6165.91	100.00%
R4A-2021-2750	11927	759891	19113.55	100.00%
R4A-2021-2849	11927	318432	8009.52	100.00%
R4A-2021-2999	11927	387594	9749.16	100.00%
R4A-2021-3000	11927	197621	4970.76	100.00%
R4A-2021-3398	11927	926519	23304.75	100.00%
R4A-2021-4119	11927	547006	13758.85	100.00%
R4A-2021-4446	11927	1284319	32304.49	100.00%
R4A-2022-40	11927	455527	11457.88	100.00%
R4A-2022-93	11927	726575	18275.55	100.00%
R4A-2022-98	11927	345374	8687.20	100.00%
R4A-2022-99	11927	915615	23030.48	100.00%
R4A-2022-104	11927	1175840	29575.92	100.00%
R4A-2022-171	11927	468738	11790.17	100.00%
R4A-2022-203	11928	908930	22860.41	100.00%
R4A-2022-210	11927	599265	15073.32	100.00%
R4A-2022-234	11927	852668	21447.17	100.00%
R4A-2022-252	11927	289358	7278.23	100.00%
R4A-2022-261	11928	1128754	28389.19	100.00%
R4A-2022-408	11927	436640	10982.81	100.00%
R4A-2022-577	11927	1080855	27186.76	100.00%
R4B-2019-5816	11927	398216	10016.33	100.00%
R4B-2021-058	11927	1374214	34565.62	100.00%
R4B-2021-060	11927	63407	1594.88	100.00%
R4B-2021-061	11927	602933	15165.58	100.00%
R4B-2021-067	11927	394058	9911.75	100.00%
R4B-2021-070	11927	239159	6015.57	100.00%
R4B-2022-00001	11926	415788	10459.20	99.99%
R4B-2022-00002	11926	1204401	30296.86	99.99%
R4B-2022-00004	11927	48002	1207.39	100.00%

R4B-2022-00005	11926	1659474	41744.27	99.99%	
R4B-2022-00006	11926	1225279	30822.04	99.99%	
R4B-2022-00007	11927	578198	14543.42	100.00%	
R4B-2022-00008	11927	465549	11709.96	100.00%	
R4B-2022-00010	11927	608279	15300.05	100.00%	
R4B-2022-00011	11927	398192	10015.73	100.00%	
R5-2018-0051	11924	90855	2285.85	99.97%	
R5-2018-0067	11924	68013	1711.16	99.97%	
R5-2018-0069	11924	173354	4361.47	99.97%	
R5-2018-0070	11924	20774	522.66	99.97%	
R5-2018-0151	11924	43169	1086.10	99.97%	
R5-2018-0219	11924	898853	22614.55	99.97%	
R5-2018-0259	11924	138480	3484.07	99.97%	
R5-2018-0334	11924	73497	1849.14	99.97%	
R5-2019-0064	11924	165720	4169.41	99.97%	
R5-2021-0041	11924	2674200	67281.11	99.97%	
R5-2021-0042	11924	342404	8614.66	99.97%	
R5-2021-0069	11924	2701457	67966.88	99.97%	
R5-2021-0092	11924	592061	14895.87	99.97%	
R5-2021-0093	11924	448963	11295.61	99.97%	
R5-2021-0137	2606	1	0.12	21.85%	failure
R5-2021-0138	11924	369753	9302.74	99.97%	
R5-2021-0139	11924	337053	8480.03	99.97%	
R5-2021-0140	11924	303288	7630.53	99.97%	
R5-2021-0142	11924	641792	16147.06	99.97%	
R5-2021-0276	11924	820846	20651.95	99.97%	
R5-2021-0277	249	2	2.41	2.09%	failure
R5-2021-0278	11924	317064	7977.12	99.97%	
R5-2021-0328	11924	1119887	28175.62	99.97%	
R5-2021-0329	11924	580254	14598.81	99.97%	
R5-2021-0334	11924	38628	971.86	99.97%	
R5-2021-0335	11924	241459	6074.95	99.97%	
R5-2021-0376	11924	132949	3344.91	99.97%	
R5-2021-0404	11922	2029	51.06	99.96%	
R5-2021-0423	776	8	3.09	6.51%	failure
R5-2021-0424	11924	474316	11933.48	99.97%	
R5-2021-0426	11924	773966	19472.48	99.97%	
R5-2021-0445	11924	386370	9720.82	99.97%	
R5-2021-0446	11924	1010259	25417.45	99.97%	
R5-2021-0447	11925	102951	2589.96	99.98%	
R5-2021-0470	11924	390884	9834.38	99.97%	
R5-2021-0472	11924	30150	758.55	99.97%	
R5-2021-0475	11924	418759	10535.70	99.97%	
R5-2021-0478	11924	901789	22688.42	99.97%	

R5-2021-0517	11924	387410	9746.98	99.97%
R6-2019-1399	11922	205124	5161.65	99.96%
R6-2019-2184	11928	262448	6600.80	100.00%
R6-2019-2722	11928	241531	6074.72	100.00%
R6-2019-2940	11928	797777	20064.81	100.00%
R6-2019-3303	11928	1603330	40325.20	100.00%
R6-2019-3499	11928	1359182	34184.66	100.00%
R6-2021-4431	11928	1417860	35660.46	100.00%
R6-2021-4446	11928	798045	20071.55	100.00%
R6-2021-4447	11928	642518	16159.91	100.00%
R6-2021-4511	11925	856428	21545.36	99.98%
R6-2021-4568	11928	393335	9892.73	100.00%
R6-2021-4599	11928	731028	18386.02	100.00%
R6-2021-4600	11928	609022	15317.45	100.00%
R6-2021-4702	11928	68384	1719.92	100.00%
R6-2021-4703	11928	283028	7118.41	100.00%
R6-2021-4711	11928	1129537	28408.88	100.00%
R6-2021-4767	11928	698814	17575.80	100.00%
R6-2021-4768	11928	372680	9373.24	100.00%
R6-2021-4769	11928	230923	5807.92	100.00%
R6-2021-4770	11928	464288	11677.26	100.00%
R6-2021-4798	11928	285003	7168.08	100.00%
R6-2021-4965	11928	1300806	32716.45	100.00%
R6-2021-5932	11925	674325	16964.15	99.98%
R6-2021-5961	11925	466808	11743.60	99.98%
R6-2021-5964	11925	542115	13638.11	99.98%
R7-2021-0149	11928	121987	3068.08	100.00%
R7-2021-0150	11922	212071	5336.46	99.96%
R7-2021-0151	11923	118448	2980.32	99.97%
R7-2021-0153	11928	385886	9705.38	100.00%
R7-2021-0155	11923	329042	8279.17	99.97%
R7-2021-0159	11928	436857	10987.35	100.00%
R7-2021-0160	11923	1202371	30253.40	99.97%
R7-2021-0161	11924	639416	16087.29	99.97%
R7-2021-0162	11922	912903	22971.89	99.96%
R7-2021-0163	11928	425604	10704.33	100.00%
R7-2021-0167	11923	477752	12020.93	99.97%
R7-2021-0168	11923	624518	15713.78	99.97%
R7-2022-0001	11928	441165	11095.70	100.00%
R7-2022-0003	11923	284306	7153.55	99.97%
R7-2022-0004	11923	568477	14303.71	99.97%
R7-2022-0007	11924	277329	6977.42	99.97%
R7-2022-0008	11928	281821	7088.05	100.00%
R7-2022-0009	11922	559008	14066.63	99.96%

R7-2022-0010	11923	356778	8977.05	99.97%	
R7-0095	11928	2640473	66410.29	100.00%	
R7-0106	11928	3823523	96165.07	100.00%	
R7-0185	11928	1937352	48726.16	100.00%	
R7-0201	11928	1387474	34896.23	100.00%	
R7-0203	11928	818914	20596.43	100.00%	
R7-0205	11928	2460579	61885.79	100.00%	
R8-2021-010	11928	359763	9048.37	100.00%	
R8-2021-011	11928	479523	12060.44	100.00%	
R8-2021-015	11928	148718	3740.39	100.00%	
R8-2021-019	11926	34252	861.61	99.99%	
R8-2021-020	11926	291049	7321.37	99.99%	
R8-2021-021	11928	123127	3096.76	100.00%	
R8-2021-024	11928	38694	973.19	100.00%	
R8-2021-025	11922	27334	687.82	99.96%	
R9-2019-074	272	0	0.00	2.28%	failure
R9-2019-100	0	0	0.00	0.00%	failure
R9-2019-102	988	2	0.61	8.28%	failure
R9-2019-154	204	0	0.00	1.71%	failure
R9-2019-170	237	0	0.00	1.99%	failure
R9-2019-311	11924	726309	18273.46	99.97%	
R9-2021-012	505	4	2.38	4.23%	failure
R9-2021-041	11928	353975	8902.79	100.00%	
R9-2021-074	11926	625168	15726.18	99.99%	
R9-2021-075	11927	330067	8302.18	100.00%	
R9-2021-078	11927	694515	17469.15	100.00%	
R9-2021-089	11927	707342	17791.78	100.00%	
R9-2021-090	11928	399298	10042.71	100.00%	
R9-2021-098	0	0	0.00	0.00%	failure
R9-2021-105	11925	347722	8747.72	99.98%	
R10-2019-669	11924	113036	2843.91	99.97%	
R10-2019-752	11925	1088296	27378.52	99.98%	
R10-2019-753	11926	403553	10151.43	99.99%	
R10-2019-777	11925	408031	10264.93	99.98%	
R10-2019-810	11926	466062	11723.85	99.99%	
R10-2019-818	11925	768076	19322.67	99.98%	
R10-2019-819	11925	1166591	29348.20	99.98%	
R10-2019-823	11924	2293616	57705.87	99.97%	
R10-2019-887	11924	1665105	41892.95	99.97%	
R10-2019-892	11927	1028117	25860.24	100.00%	
R10-2019-907	11925	2852511	71761.28	99.98%	
R10-2019-908	11927	677731	17046.98	100.00%	
R10-2019-909	11925	86017	2163.95	99.98%	
R10-2019-950	11924	587117	14771.48	99.97%	

R10-2019-951	11925	1170346	29442.67	99.98%	
R10-2019-970	11925	3165433	79633.53	99.98%	
R10-2021-001	11925	694922	17482.31	99.98%	
R10-2021-002	11925	221931	5583.17	99.98%	
R10-2021-003	11926	61216	1539.90	99.99%	
R10-2021-004	11926	154648	3890.19	99.99%	
R10-2021-005	11924	407426	10250.57	99.97%	
R10-2021-006	11925	62249	1566.01	99.98%	
R10-2021-007	11926	305952	7696.26	99.99%	
R10-2021-008	11924	146694	3690.72	99.97%	
R10-2021-009	11924	297119	7475.32	99.97%	
R10-2021-010	11925	86223	2169.13	99.98%	
R11-2021-02	11925	1412	35.52	99.98%	
R11-2021-58	11928	1279	32.17	100.00%	
R11-2021-61	11925	718	18.06	99.98%	
R11-2021-68	11926	667	16.78	99.99%	
R11-2022-07	11924	1128633	28395.66	99.97%	
R11-2022-11	11924	727611	18306.21	99.97%	
R11-2022-14	11924	180282	4535.78	99.97%	
R11-2022-15	11927	131811	3315.44	100.00%	
R11-2022-16	11926	207064	5208.72	99.99%	
R11-2022-19	11926	445709	11211.86	99.99%	
R11-2022-25	11927	613537	15432.30	100.00%	
R11-2022-28	11924	341978	8603.94	99.97%	
R11-2022-30	11924	871493	21926.19	99.97%	
R11-2022-31	1872	14	2.24	15.70%	failure
R11-2022-32	11925	558307	14045.46	99.98%	
R11-2022-33	11927	332530	8364.13	100.00%	
R12-2019-0014	11925	5316	133.74	99.98%	
R12-2019-0472	11925	180351	4537.13	99.98%	
R12-2019-0521	11925	308509	7761.23	99.98%	
R12-2019-0538	11925	186539	4692.81	99.98%	
R12-2019-0611	11927	410381	10322.32	100.00%	
R12-2019-0637	11925	920324	23152.81	99.98%	
R12-2019-0684	11927	284658	7160.01	100.00%	
R12-2019-0699	11925	108386	2726.69	99.98%	
R12-2019-0861	11928	757441	19050.33	100.00%	
R12-2021-020	11925	90417	2274.64	99.98%	
R12-2021-033	11928	689163	17333.07	100.00%	
R12-2021-047	11927	317263	7980.12	100.00%	
R12-2021-063	11925	853	21.46	99.98%	
R12-2021-100	11928	113946	2865.85	100.00%	
R12-2021-175	11926	54949	1382.25	99.99%	
R12-2021-176	11925	138880	3493.84	99.98%	

R12-2021-232	11927	481555	12112.56	100.00%	
R12-2021-237	11927	1382732	34779.88	100.00%	
R12-2021-244	11927	27144	682.75	100.00%	
R12-2021-280	11927	328937	8273.76	100.00%	
R12-2021-339	11927	226126	5687.75	100.00%	
R12-2021-384	11928	87400	2198.19	100.00%	
R12-2021-905	11926	338906	8525.22	99.99%	
R12-2021-954	11927	1232674	31005.47	100.00%	
R12-2021-1086	11926	94645	2380.81	99.99%	
R12-2021-1087	11926	330149	8304.94	99.99%	
R13-2021-617	11924	64482	1622.32	99.97%	
R13-2021-1490	2685	74	8.27	22.51%	failure
R13-2021-1498	11926	649389	16335.46	99.99%	
R13-2021-1745	11926	435552	10956.36	99.99%	
R13-2021-1748	11926	793025	19948.64	99.99%	
R13-2021-2121	11925	558149	14041.48	99.98%	
R13-2021-2131	11928	49076	1234.31	100.00%	
R13-2021-2136	11926	585633	14731.67	99.99%	
R13-2021-2146	11926	62338	1568.12	99.99%	
R13-2021-2155	11926	1121220	28204.43	99.99%	
