# Supplementary material for: Phylogeography Analysis Reveals Rabies Epidemiology, Evolution, and Transmission in the Philippines
Source: Mol Biol Evol. 2025 Feb 12;42(2):msaf007. doi: 10.1093/molbev/msaf007 (PMC11815495; doi:10.1093/molbev/msaf007)
Supplement: msaf007_Supplementary_Data [file msaf007_supplementary_data.zip › Supplementary Table 3.pdf]

Supplementary table S3. Information of 353 RABV strains with their full genomes sequenced in our study.

Sample_id	Collection time	Location	Host	Genotype	Acceccsion No.
CAR-2019-085	26-Aug-2019	Kalinga	Canis lupus familiaris	SEA4-L	OR971323
CAR-2019-092	4-Sep-2019	Benguet	Canis lupus familiaris	SEA4-L	OR971324
CAR-2019-098	16-Sep-2019	Kalinga	Canis lupus familiaris	SEA4-L	OR971325
CAR-2019-099	26-Sep-2019	Benguet	Canis lupus familiaris	SEA4-L	OR971326
CAR-2019-103	23-Sep-2019	Kalinga	Canis lupus familiaris	SEA4-L	OR971327
CAR-2019-111	14-Oct-2019	Abra	Canis lupus familiaris	SEA4-L	OR971328
CAR-2019-208	28-Oct-2019	Kalinga	Canis lupus familiaris	SEA4-L	OR971329
CAR-2021-015	24-Feb-2021	Abra	Canis lupus familiaris	SEA4-L	OR971330
CAR-2021-016	28-Feb-2021	Abra	Canis lupus familiaris	SEA4-L	OR971331
CAR-2021-022	11-Mar-2021	Ifugao	Canis lupus familiaris	SEA4-L	OR971332
CAR-2021-032	3-May-2021	Apayao	Canis lupus familiaris	SEA4-L	OR971333
CAR-2021-033	10-May-2021	Abra	Canis lupus familiaris	SEA4-L	OR971334
CAR-2021-034	14-May-2021	Apayao	Canis lupus familiaris	SEA4-L	OR971335
CAR-2021-040	6-Aug-2021	Abra	Canis lupus familiaris	SEA4-L	OR971336
CAR-2021-044	18-Jun-2021	Abra	Canis lupus familiaris	SEA4-L	OR971337
CAR-2021-046	25-Jun-2021	Benguet	Canis lupus familiaris	SEA4-L	OR971338
CAR-2021-074	11-Mar-2021	Abra	Canis lupus familiaris	SEA4-L	OR971339
CAR-2021-075	15-Nov-2021	Abra	Canis lupus familiaris	SEA4-L	OR971340
CAR-2022-001	1-Apr-2022	Abra	Canis lupus familiaris	SEA4-L	OR971341
CAR-2022-002	1-Jun-2022	Mountain	Canis lupus familiaris	SEA4-L	OR971342
CAR-2022-004	14-Jan-2022	Abra	Canis lupus familiaris	SEA4-L	OR971343
CAR-2022-007	2-Jan-2022	Abra	Canis lupus familiaris	SEA4-L	OR971344
CAR-2022-018	3-Oct-2022	Mountain	Canis lupus familiaris	SEA4-L	OR971345
CAR-2022-020	16-Mar-2022	Abra	Canis lupus familiaris	SEA4-L	OR971346

NCR-2019-6154	5-Sep-2019	Metro Manila	Canis lupus familiaris	SEA4-L	OR971347
NCR-2019-6544	24-Sep-2019	Metro Manila	Canis lupus familiaris	SEA4-L	OR971348
NCR-2019-6661	30-Sep-2019	Metro Manila	Canis lupus familiaris	SEA4-L	OR971349
NCR-2020-1676	5-Feb-2020	Metro Manila	Canis lupus familiaris	SEA4-L	OR971350
NCR-2020-2509	31-Mar-2020	Metro Manila	Canis lupus familiaris	SEA4-L	OR971351
NCR-2020-2625	15-Jun-2020	Metro Manila	Canis lupus familiaris	SEA4-L	OR971352
NCR-2020-2865	23-Jul-2020	Metro Manila	Canis lupus familiaris	SEA4-L	OR971353
NCR-2020-3068	24-Aug-2020	Metro Manila	Canis lupus familiaris	SEA4-L	OR971354
NCR-2020-3257	21-Sep-2020	Metro Manila	Canis lupus familiaris	SEA4-L	OR971355
NCR-2020-3323	10-Jan-2020	Metro Manila	Canis lupus familiaris	SEA4-L	OR971356
NCR-2021-3600	10-Jul-2021	Metro Manila	Canis lupus familiaris	SEA4-L	OR971357
NCR-2021-3654	13-Oct-2021	Metro Manila	Canis lupus familiaris	SEA4-L	OR971358
NCR-2021-3901	11-Feb-2021	Metro Manila	Canis lupus familiaris	SEA4-L	OR971359
NCR-2021-4011	11-May-2021	Metro Manila	Canis lupus familiaris	SEA4-L	OR971360
NCR-2022-1945	10-Jun-2022	Metro Manila	Canis lupus familiaris	SEA4-L	OR971361
NCR-2022-1961	13-Jun-2022	Metro Manila	Canis lupus familiaris	SEA4-L	OR971362
NCR-2022-2199	27-Jun-2022	Metro Manila	Canis lupus familiaris	SEA4-L	OR971363
NCR-2022-2209	28-Jun-2022	Metro Manila	Canis lupus familiaris	SEA4-L	OR971364
NCR-2022-2610	21-Jul-2022	Metro Manila	Canis lupus familiaris	SEA4-L	OR971365
NCR-2022-2837	5-Aug-2022	Metro Manila	Canis lupus familiaris	SEA4-L	OR971366
NCR-2022-2918	9-Aug-2022	Metro Manila	Canis lupus familiaris	SEA4-L	OR971367
NCR-2022-2923	9-Aug-2022	Metro Manila	Felis catus	SEA4-L	OR971368
NCR-2022-2994	15-Aug-2022	Metro Manila	Canis lupus familiaris	SEA4-L	OR971369
NCR-2022-2995	15-Aug-2022	Metro Manila	Canis lupus familiaris	SEA4-L	OR971370
NCR-2022-3041	18-Aug-2022	Metro Manila	Felis catus	SEA4-L	OR971371
NCR-2022-3089	22-Aug-2022	Metro Manila	Canis lupus familiaris	SEA4-L	OR971372

NCR-2022-3192	26-Aug-2022	Metro Manila	Canis lupus familiaris	SEA4-L	OR971373
NCR-2022-3197	26-Aug-2022	Metro Manila	Canis lupus familiaris	SEA4-L	OR971374
NCR-2022-3207	30-Aug-2022	Metro Manila	Canis lupus familiaris	SEA4-L	OR971375
NCR-2022-3281	5-Sep-2022	Metro Manila	Canis lupus familiaris	SEA4-L	OR971376
NCR-2022-3357	8-Sep-2022	Metro Manila	Canis lupus familiaris	SEA4-L	OR971377
NCR-2022-3487	16-Sep-2022	Metro Manila	Canis lupus familiaris	SEA4-L	OR971378
NCR-2022-3580	23-Sep-2022	Metro Manila	Canis lupus familiaris	SEA4-L	OR971379
NCR-2022-3616	28-Sep-2022	Metro Manila	Canis lupus familiaris	SEA4-L	OR971380
R1-2019-178	10-Sep-2019	Pangasinan	Canis lupus familiaris	SEA4-L	OR971381
R1-2019-193	1-Oct-2019	Pangasinan	Canis lupus familiaris	SEA4-L	OR971382
R1-2019-196	15-Oct-2019	Pangasinan	Canis lupus familiaris	SEA4-L	OR971383
R1-2019-204	21-Oct-2019	Pangasinan	Canis lupus familiaris	SEA4-L	OR971384
R1-2019-212	6-Nov-2019	Ilocos Sur	Canis lupus familiaris	SEA4-L	OR971385
R1-2021-161	28-Oct-2021	Pangasinan	Canis lupus familiaris	SEA4-L	OR971386
R1-2021-162	29-Oct-2021	Pangasinan	Felis catus	SEA4-L	OR971387
R1-2021-165	11-Feb-2021	La Union	Canis lupus familiaris	SEA4-L	OR971388
R1-2021-166	11-Aug-2021	Pangasinan	Canis lupus familiaris	SEA4-L	OR971389
R1-2022-034	4-Apr-2022	La Union	Canis lupus familiaris	SEA4-L	OR971390
R1-2022-040	18-Apr-2022	Pangasinan	Canis lupus familiaris	SEA4-L	OR971391
R1-2022-042	21-Apr-2022	Pangasinan	Canis lupus familiaris	SEA4-L	OR971392
R2-2019-7027	20-Aug-2019	Cagayan	Canis lupus familiaris	SEA4-L	OR971393
R2-2019-7465	-	Pangasinan	Canis lupus familiaris	SEA4-L	OR971394
R2-2019-7838	20-Sep-2019	Nueva Vizcaya	Canis lupus familiaris	SEA4-L	OR971395
R2-2019-8088	4-Oct-2019	Isabela	Canis lupus familiaris	SEA4-L	OR971396
R2-2019-8166	7-Oct-2019	Nueva Vizcaya	Canis lupus familiaris	SEA4-L	OR971397
R2-2019-8204	8-Oct-2019	Isabela	Canis lupus familiaris	SEA4-L	OR971398

R2-2019-8228	10-Oct-2019	Isabela	Canis lupus familiaris	SEA4-L	OR971399
R2-2021-082	8-Sep-2021	Isabela	Canis lupus familiaris	SEA4-L	OR971400
R2-2021-083	8-Oct-2021	Nueva Vizcaya	Canis lupus familiaris	SEA4-L	OR971401
R2-2021-084	13-Aug-2021	Cagayan	Canis lupus familiaris	SEA4-L	OR971402
R2-2021-087	17-Aug-2021	Nueva Vizcaya	Canis lupus familiaris	SEA4-L	OR971403
R2-2021-106	11-May-2021	Isabela	Canis lupus familiaris	SEA4-L	OR971404
R2-2021-121	20-Dec-2021	Isabela	Canis lupus familiaris	SEA4-L	OR971405
R2-2021-124	27-Dec-2021	Cagayan	Canis lupus familiaris	SEA4-L	OR971406
R2-2021-125	30-Dec-2021	Cagayan	Canis lupus familiaris	SEA4-L	OR971407
R3-2019-5590	8-Feb-2019	Bulacan	Canis lupus familiaris	SEA4-L	OR971408
R3-2019-5829	20-Aug-2019	Bulacan	Canis lupus familiaris	SEA4-L	OR971409
R3-2019-5868	20-Aug-2019	Bulacan	Canis lupus familiaris	SEA4-L	OR971410
R3-2019-6317	13-Sep-2019	Bulacan	Canis lupus familiaris	SEA4-L	OR971411
R3-2021-0274	17-Mar-2021	Bataan	Canis lupus familiaris	SEA4-L	OR971412
R3-2021-0282	5-Apr-2021	Nueva Ecija	Canis lupus familiaris	SEA4-L	OR971413
R3-2021-0286	7-Apr-2021	Nueva Ecija	Canis lupus familiaris	SEA4-L	OR971414
R3-2021-0289	13-Apr-2021	Pampanga	Canis lupus familiaris	SEA4-L	OR971415
R3-2021-0294	16-Apr-2021	Pampanga	Canis lupus familiaris	SEA4-L	OR971416
R3-2021-0296	16-Apr-2021	Pampanga	Canis lupus familiaris	SEA4-L	OR971417
R3-2021-0300	26-Apr-2021	Pampanga	Canis lupus familiaris	SEA4-L	OR971418
R3-2021-0304	5-May-2021	Pampanga	Canis lupus familiaris	SEA4-L	OR971419
R3-2021-0322	24-May-2021	Bulacan	Canis lupus familiaris	SEA4-L	OR971420
R3-2021-0467	31-Aug-2021	Pampanga	Canis lupus familiaris	SEA4-L	OR971421
R3-2021-0468	31-Aug-2021	Nueva Ecija	Canis lupus familiaris	SEA4-L	OR971422
R3-2021-0469	31-Aug-2021	Bulacan	Canis lupus familiaris	SEA4-L	OR971423
R3-2021-0472	9-Mar-2021	Pampanga	Canis lupus familiaris	SEA4-L	OR971424

R3-2021-0483	9-Sep-2021	Bulacan	Canis lupus familiaris	SEA4-L	OR971425
R3-2021-0484	9-Sep-2021	Bulacan	Canis lupus familiaris	SEA4-L	OR971426
R3-2021-0487	9-Oct-2021	Pampanga	Canis lupus familiaris	SEA4-L	OR971427
R3-2021-0495	24-Sep-2021	Pampanga	Canis lupus familiaris	SEA4-L	OR971428
R3-2021-0498	10-Apr-2021	Bulacan	Canis lupus familiaris	SEA4-L	OR971429
R3-2021-0501	10-May-2021	Bataan	Canis lupus familiaris	SEA4-L	OR971430
R3-2021-0502	10-Jul-2021	Pampanga	Canis lupus familiaris	SEA4-L	OR971431
R3-2021-0504	10-Aug-2021	Pampanga	Canis lupus familiaris	SEA4-L	OR971432
R3-2021-0513	13-Oct-2021	Bataan	Canis lupus familiaris	SEA4-L	OR971433
R3-2021-0514	13-Oct-2021	Zambales	Felis catus	SEA4-L	OR971434
R3-2021-0543	27-Oct-2021	Bulacan	Canis lupus familiaris	SEA4-L	OR971435
R3-2021-0731	12-Sep-2021	Nueva Ecija	Canis lupus familiaris	SEA4-L	OR971436
R3-2021-0753	22-Dec-2021	Nueva Ecija	Canis lupus familiaris	SEA4-L	OR971437
R3-2021-3790	25-Oct-2021	Bulacan	Canis lupus familiaris	SEA4-L	OR971438
R3-2022-0002	1-Mar-2022	Pampanga	Canis lupus familiaris	SEA4-L	OR971439
R3-2022-0004	1-Apr-2022	Pampanga	Canis lupus familiaris	SEA4-L	OR971440
R3-2022-0006	18-Jan-2022	Nueva Ecija	Canis lupus familiaris	SEA4-L	OR971441
R3-2022-0013	1-Jul-2022	Zambales	Canis lupus familiaris	SEA4-L	OR971442
R3-2022-0014	1-Oct-2022	Pampanga	Canis lupus familiaris	SEA4-L	OR971443
R3-2022-0016	1-Nov-2022	Bulacan	Canis lupus familiaris	SEA4-L	OR971444
R3-2022-0017	13-Jan-2022	Bulacan	Canis lupus familiaris	SEA4-L	OR971445
R3-2022-0019	18-Jan-2022	Pampanga	Canis lupus familiaris	SEA4-L	OR971446
R4A-2019-472	6-May-2019	Batangas	Canis lupus familiaris	SEA4-L	OR971447
R4A-2019-772	1-Aug-2019	Batangas	Canis lupus familiaris	SEA4-GrSL	OR971448
R4A-2019-857	27-Aug-2019	Quezon	Canis lupus familiaris	SEA4-L	OR971449
R4A-2019-892	9-Sep-2019	Batangas	Canis lupus familiaris	SEA4-L	OR971450

R4A-2019-949	25-Sep-2019	Laguna	Canis lupus familiaris	SEA4-L	OR971451
R4A-2019-989	3-Oct-2019	Laguna	Canis lupus familiaris	SEA4-L	OR971452
R4A-2019-1023	17-Oct-2019	Quezon	Canis lupus familiaris	SEA4-L	OR971453
R4A-2019-1033	24-Oct-2019	Quezon	Canis lupus familiaris	SEA4-L	OR971454
R4A-2019-5622	5-Aug-2019	Rizal	Canis lupus familiaris	SEA4-L	OR971455
R4A-2019-5970	27-Aug-2019	Rizal	Canis lupus familiaris	SEA4-L	OR971456
R4A-2020-0084	3-Jan-2020	Rizal	Canis lupus familiaris	SEA4-L	OR971457
R4A-2020-2377	4-Mar-2020	Rizal	Canis lupus familiaris	SEA4-L	OR971458
R4A-2020-2577	29-May-2020	Rizal	Canis lupus familiaris	SEA4-L	OR971459
R4A-2021-1574	10-Dec-2021	Batangas	Canis lupus familiaris	SEA4-L	OR971460
R4A-2021-1668	29-Nov-2021	Quezon	Canis lupus familiaris	SEA4-L	OR971461
R4A-2021-2633	27-Jul-2021	Rizal	Canis lupus familiaris	SEA4-L	OR971462
R4A-2021-2750	8-May-2021	Rizal	Canis lupus familiaris	SEA4-L	OR971463
R4A-2021-2849	8-Nov-2021	Rizal	Canis lupus familiaris	SEA4-L	OR971464
R4A-2021-2999	26-Aug-2021	Rizal	Canis lupus familiaris	SEA4-L	OR971465
R4A-2021-3000	26-Aug-2021	Rizal	Canis lupus familiaris	SEA4-L	OR971466
R4A-2021-3398	23-Sep-2021	Rizal	Canis lupus familiaris	SEA4-L	OR971467
R4A-2021-4119	11-Nov-2021	Rizal	Canis lupus familiaris	SEA4-L	OR971468
R4A-2021-4446	12-Jan-2021	Rizal	Canis lupus familiaris	SEA4-L	OR971469
R4A-2022-40	1-Nov-2022	Batangas	Canis lupus familiaris	SEA4-L	OR971470
R4A-2022-93	21-Jan-2022	Quezon	Canis lupus familiaris	SEA4-L	OR971471
R4A-2022-98	24-Jan-2022	Quezon	Canis lupus familiaris	SEA4-L	OR971472
R4A-2022-99	25-Jan-2022	Quezon	Canis lupus familiaris	SEA4-L	OR971473
R4A-2022-104	25-Jan-2022	Batangas	Canis lupus familiaris	SEA4-L	OR971474
R4A-2022-171	2-Jul-2022	Cavite	Canis lupus familiaris	SEA4-L	OR971475
R4A-2022-203	14-Feb-2022	Quezon	Canis lupus familiaris	SEA4-L	OR971476

R4A-2022-210	15-Feb-2022	Quezon	Canis lupus familiaris	SEA4-L	OR971477
R4A-2022-234	17-Feb-2022	Quezon	Canis lupus familiaris	SEA4-L	OR971478
R4A-2022-252	22-Feb-2022	Laguna	Canis lupus familiaris	SEA4-L	OR971479
R4A-2022-261	24-Feb-2022	Cavite	Canis lupus familiaris	SEA4-L	OR971480
R4A-2022-408	3-Oct-2022	Quezon	Canis lupus familiaris	SEA4-L	OR971481
R4A-2022-577	24-Mar-2022	Quezon	Canis lupus familiaris	SEA4-L	OR971482
R4B-2019-5816	16-Aug-2019	Oriental Mindoro	Canis lupus familiaris	SEA4-L	OR971483
R4B-2021-058	2-Sep-2021	Oriental Mindoro	Canis lupus familiaris	SEA4-L	OR971484
R4B-2021-060	3-May-2021	Oriental Mindoro	Canis lupus familiaris	SEA4-L	OR971485
R4B-2021-061	3-Oct-2021	Oriental Mindoro	Canis lupus familiaris	SEA4-L	OR971486
R4B-2021-067	14-Apr-2021	Oriental Mindoro	Canis lupus familiaris	SEA4-L	OR971487
R4B-2021-070	23-Apr-2021	Oriental Mindoro	Canis lupus familiaris	SEA4-L	OR971488
R4B-2022-00001	5-Jan-2022	Oriental Mindoro	Canis lupus familiaris	SEA4-L	OR971489
R4B-2022-00002	14-Jan-2022	Oriental Mindoro	Canis lupus familiaris	SEA4-L	OR971490
R4B-2022-00004	4-Feb-2022	Occidental Mindoro	Canis lupus familiaris	SEA4-L	OR971491
R4B-2022-00005	2-Sep-2022	Oriental Mindoro	Canis lupus familiaris	SEA4-L	OR971492
R4B-2022-00006	9-Feb-2022	Oriental Mindoro	Canis lupus familiaris	SEA4-L	OR971493
R4B-2022-00007	16-Feb-2022	Oriental Mindoro	Canis lupus familiaris	SEA4-L	OR971494
R4B-2022-00008	17-Feb-2022	Oriental Mindoro	Canis lupus familiaris	SEA4-L	OR971495
R4B-2022-00010	3-Mar-2022	Oriental Mindoro	Canis lupus familiaris	SEA4-L	OR971496
R4B-2022-00011	-	Oriental Mindoro	Canis lupus familiaris	SEA4-L	OR971497
R5-2018-0051	8-Feb-2018	Sorsogon	Canis lupus familiaris	SEA4-L	OR971498
R5-2018-0067	20-Feb-2018	Sorsogon	Canis lupus familiaris	SEA4-L	OR971499
R5-2018-0069	27-Feb-2018	Sorsogon	Canis lupus familiaris	SEA4-L	OR971500
R5-2018-0070	27-Feb-2018	Sorsogon	Canis lupus familiaris	SEA4-L	OR971501
R5-2018-0151	2-May-2018	Sorsogon	Bos taurus	SEA4-L	OR971502

R5-2018-0219	10-Aug-2018	Sorsogon	Canis lupus familiaris	SEA4-L	OR971503
R5-2018-0259	14-Sep-2018	Sorsogon	Canis lupus familiaris	SEA4-L	OR971504
R5-2018-0334	7-Dec-2018	Camarines Sur	Canis lupus familiaris	SEA4-L	OR971505
R5-2019-0064	7-Mar-2019	Camarines Sur	Canis lupus familiaris	SEA4-L	OR971506
R5-2021-0041	5-Jan-2021	Albay	Canis lupus familiaris	SEA4-L	OR971507
R5-2021-0042	5-Jan-2021	Albay	Canis lupus familiaris	SEA4-L	OR971508
R5-2021-0069	11-Jan-2021	Albay	Canis lupus familiaris	SEA4-L	OR971509
R5-2021-0092	19-Jan-2021	Albay	Canis lupus familiaris	SEA4-L	OR971510
R5-2021-0093	20-Jan-2021	Albay	Canis lupus familiaris	SEA4-L	OR971511
R5-2021-0138	1-Feb-2021	Camarines Norte	Canis lupus familiaris	SEA4-L	OR971512
R5-2021-0139	1-Feb-2021	Camarines Norte	Canis lupus familiaris	SEA4-L	OR971513
R5-2021-0140	1-Feb-2021	Camarines Norte	Canis lupus familiaris	SEA4-L	OR971514
R5-2021-0142	1-Feb-2021	Camarines Sur	Canis lupus familiaris	SEA4-L	OR971515
R5-2021-0276	5-Mar-2021	Camarines Sur	Canis lupus familiaris	SEA4-L	OR971516
R5-2021-0278	5-Mar-2021	Albay	Canis lupus familiaris	SEA4-L	OR971517
R5-2021-0328	22-Mar-2021	Camarines Sur	Canis lupus familiaris	SEA4-L	OR971518
R5-2021-0329	22-Mar-2021	Camarines Sur	Canis lupus familiaris	SEA4-L	OR971519
R5-2021-0334	22-Mar-2021	Sorsogon	Canis lupus familiaris	SEA4-L	OR971520
R5-2021-0335	22-Mar-2021	Albay	Canis lupus familiaris	SEA4-L	OR971521
R5-2021-0376	2-Apr-2021	Albay	Canis lupus familiaris	SEA4-L	OR971522
R5-2021-0404	12-Apr-2021	Camarines Sur	Canis lupus familiaris	SEA4-L	OR971523
R5-2021-0424	19-Apr-2021	Camarines Norte	Canis lupus familiaris	SEA4-L	OR971524
R5-2021-0426	19-Apr-2021	Albay	Canis lupus familiaris	SEA4-L	OR971525
R5-2021-0445	26-Apr-2021	Sorsogon	Canis lupus familiaris	SEA4-L	OR971526
R5-2021-0446	27-Apr-2021	Camarines Sur	Canis lupus familiaris	SEA4-L	OR971527
R5-2021-0447	26-Apr-2021	Sorsogon	Canis lupus familiaris	SEA4-L	OR971528

R5-2021-0470	3-May-2021	Camarines Norte	Canis lupus familiaris	SEA4-L	OR971529
R5-2021-0472	3-May-2021	Camarines Norte	Canis lupus familiaris	SEA4-L	OR971530
R5-2021-0475	5-May-2021	Camarines Sur	Canis lupus familiaris	SEA4-L	OR971531
R5-2021-0478	6-May-2021	Albay	Canis lupus familiaris	SEA4-L	OR971532
R5-2021-0517	10-May-2021	Camarines Norte	Canis lupus familiaris	SEA4-L	OR971533
R6-2019-1399	15-Apr-2019	Aklan	Canis lupus familiaris	SEA4-L	OR971534
R6-2019-2184	11-Jun-2019	Iloilo	Canis lupus familiaris	SEA4-V	OR971535
R6-2019-2722	17-Jun-2019	Guimaras	Canis lupus familiaris	SEA4-V	OR971536
R6-2019-2940	29-Aug-2019	Capiz	Canis lupus familiaris	SEA4-V	OR971537
R6-2019-3303	17-Sep-2019	Antique	Canis lupus familiaris	SEA4-V	OR971538
R6-2019-3499	10-Oct-2019	Iloilo	Canis lupus familiaris	SEA4-V	OR971539
R6-2021-4431	5-Oct-2021	Iloilo	Canis lupus familiaris	SEA4-V	OR971540
R6-2021-4446	6-Oct-2021	Iloilo	Canis lupus familiaris	SEA4-V	OR971541
R6-2021-4447	6-Oct-2021	Iloilo	Canis lupus familiaris	SEA4-V	OR971542
R6-2021-4511	7-Oct-2021	Antique	Canis lupus familiaris	SEA4-L	OR971543
R6-2021-4568	12-Oct-2021	Guimaras	Canis lupus familiaris	SEA4-V	OR971544
R6-2021-4599	12-Oct-2021	Iloilo	Canis lupus familiaris	SEA4-V	OR971545
R6-2021-4600	13-Oct-2021	Iloilo	Canis lupus familiaris	SEA4-V	OR971546
R6-2021-4702	19-Oct-2021	Negros Occidental	Canis lupus familiaris	SEA4-V	OR971547
R6-2021-4703	19-Oct-2021	Iloilo	Canis lupus familiaris	SEA4-V	OR971548
R6-2021-4711	22-Oct-2021	Iloilo	Canis lupus familiaris	SEA4-V	OR971549
R6-2021-4767	25-Oct-2021	Iloilo	Canis lupus familiaris	SEA4-V	OR971550
R6-2021-4768	25-Oct-2021	Iloilo	Canis lupus familiaris	SEA4-V	OR971551
R6-2021-4769	26-Oct-2021	Iloilo	Canis lupus familiaris	SEA4-V	OR971552
R6-2021-4770	27-Oct-2021	Iloilo	Canis lupus familiaris	SEA4-V	OR971553
R6-2021-4798	29-Oct-2021	Iloilo	Canis lupus familiaris	SEA4-V	OR971554

R6-2021-4965	27-Oct-2021	Iloilo	Canis lupus familiaris	SEA4-V	OR971555
R6-2021-5932	12-Jul-2021	Guimaras	Canis lupus familiaris	SEA4-V	OR971556
R6-2021-5961	12-Sep-2021	Iloilo	Canis lupus familiaris	SEA4-V	OR971557
R6-2021-5964	12-Sep-2021	Iloilo	Canis lupus familiaris	SEA4-V	OR971558
R7-2021-0149	13-Oct-2021	Cebu	Canis lupus familiaris	SEA4-V	OR971559
R7-2021-0150	13-Oct-2021	Cebu	Canis lupus familiaris	SEA4-V	OR971560
R7-2021-0151	18-Oct-2021	Cebu	Canis lupus familiaris	SEA4-V	OR971561
R7-2021-0153	21-Oct-2021	Cebu	Canis lupus familiaris	SEA4-V	OR971562
R7-2021-0155	26-Oct-2021	Cebu	Canis lupus familiaris	SEA4-V	OR971563
R7-2021-0159	11-Sep-2021	Cebu	Canis lupus familiaris	SEA4-V	OR971564
R7-2021-0160	11-Sep-2021	Cebu	Canis lupus familiaris	SEA4-V	OR971565
R7-2021-0161	11-Sep-2021	Cebu	Canis lupus familiaris	SEA4-V	OR971566
R7-2021-0162	11-Oct-2021	Cebu	Canis lupus familiaris	SEA4-V	OR971567
R7-2021-0163	11-Dec-2021	Cebu	Canis lupus familiaris	SEA4-V	OR971568
R7-2021-0167	22-Nov-2021	Cebu	Canis lupus familiaris	SEA4-V	OR971569
R7-2021-0168	22-Nov-2021	Cebu	Canis lupus familiaris	SEA4-V	OR971570
R7-2022-0001	1-Jul-2022	Cebu	Canis lupus familiaris	SEA4-V	OR971571
R7-2022-0003	26-Jan-2022	Cebu	Canis lupus familiaris	SEA4-V	OR971572
R7-2022-0004	2-Aug-2022	Cebu	Canis lupus familiaris	SEA4-V	OR971573
R7-2022-0007	14-Feb-2022	Cebu	Canis lupus familiaris	SEA4-V	OR971574
R7-2022-0008	15-Feb-2022	Cebu	Canis lupus familiaris	SEA4-V	OR971575
R7-2022-0009	21-Feb-2022	Cebu	Canis lupus familiaris	SEA4-V	OR971576
R7-2022-0010	28-Feb-2022	Cebu	Canis lupus familiaris	SEA4-V	OR971577
R7-0095	-	Cebu	Canis lupus familiaris	SEA4-V	OR971578
R7-0106	-	Cebu	Canis lupus familiaris	SEA4-V	OR971579
R7-0185	-	Cebu	Canis lupus familiaris	SEA4-V	OR971580

R7-0201	-	Cebu	Canis lupus familiaris	SEA4-V	OR971581
R7-0203	-	Cebu	Canis lupus familiaris	SEA4-V	OR971582
R7-0205	-	Cebu	Canis lupus familiaris	SEA4-V	OR971583
R8-2021-010	22-Feb-2021	Biliran	Canis lupus familiaris	SEA4-V	OR971584
R8-2021-011	26-Feb-2021	Biliran	Canis lupus familiaris	SEA4-V	OR971585
R8-2021-015	23-Mar-2021	Biliran	Canis lupus familiaris	SEA4-V	OR971586
R8-2021-019	30-Mar-2021	Leyte	Canis lupus familiaris	SEA4-M	OR971587
R8-2021-020	7-Apr-2021	Southern Leyte	Canis lupus familiaris	SEA4-M	OR971588
R8-2021-021	15-Apr-2021	Samar	Canis lupus familiaris	SEA4-V	OR971589
R8-2021-024	16-Apr-2021	Biliran	Canis lupus familiaris	SEA4-V	OR971590
R8-2021-025	26-Apr-2021	Samar	Canis lupus familiaris	SEA4-V	OR971591
R9-2019-311	9-Oct-2019	Zamboanga Del Norte	Canis lupus familiaris	SEA4-MZ	OR971592
R9-2021-041	5-Jan-2021	Zamboanga	Canis lupus familiaris	SEA4-M	OR971593
R9-2021-074	20-Aug-2021	Zamboanga del Sur	Canis lupus familiaris	SEA4-M	OR971594
R9-2021-075	23-Aug-2021	Zamboanga del Sur	Canis lupus familiaris	SEA4-M	OR971595
R9-2021-078	27-Aug-2021	Zamboanga	Canis lupus familiaris	SEA4-M	OR971596
R9-2021-089	10-Jul-2021	Zamboanga Sibugay	Canis lupus familiaris	SEA4-M	OR971597
R9-2021-090	10-Dec-2021	Zamboanga del Sur	Canis lupus familiaris	SEA4-M	OR971598
R9-2021-105	12-Sep-2021	Zamboanga Del Norte	Canis lupus familiaris	SEA4-M	OR971599
R10-2019-669	12-Sep-2019	Misamis Oriental	Canis lupus familiaris	SEA4-M	OR971600
R10-2019-752	11-Oct-2019	Bukidnon	Canis lupus familiaris	SEA4-M	OR971601
R10-2019-753	14-Oct-2019	Misamis Oriental	Canis lupus familiaris	SEA4-M	OR971602
R10-2019-777	21-Oct-2019	Iligan	Canis lupus familiaris	SEA4-M	OR971603
R10-2019-810	7-Nov-2019	Bukidnon	Canis lupus familiaris	SEA4-M	OR971604
R10-2019-818	8-Nov-2019	Iligan	Canis lupus familiaris	SEA4-M	OR971605
R10-2019-819	9-Nov-2019	Bukidnon	Canis lupus familiaris	SEA4-M	OR971606

R10-2019-823	11-Nov-2019	Misamis Oriental	Canis lupus familiaris	SEA4-M	OR971607
R10-2019-887	25-Nov-2019	Misamis Oriental	Canis lupus familiaris	SEA4-M	OR971608
R10-2019-892	28-Nov-2019	Bukidnon	Canis lupus familiaris	SEA4-M	OR971609
R10-2019-907	4-Dec-2019	Misamis Oriental	Canis lupus familiaris	SEA4-M	OR971610
R10-2019-908	4-Dec-2019	Iligan	Canis lupus familiaris	SEA4-M	OR971611
R10-2019-909	4-Dec-2019	Iligan	Canis lupus familiaris	SEA4-M	OR971612
R10-2019-950	16-Dec-2019	Misamis Oriental	Canis lupus familiaris	SEA4-M	OR971613
R10-2019-951	16-Dec-2019	Misamis Oriental	Canis lupus familiaris	SEA4-M	OR971614
R10-2019-970	27-Dec-2019	Misamis Oriental	Canis lupus familiaris	SEA4-M	OR971615
R10-2021-001	2-Sep-2021	Misamis Oriental	Canis lupus familiaris	SEA4-M	OR971616
R10-2021-002	2-Sep-2021	Misamis Oriental	Canis lupus familiaris	SEA4-M	OR971617
R10-2021-003	23-Aug-2021	Bukidnon	Canis lupus familiaris	SEA4-M	OR971618
R10-2021-004	20-Aug-2021	Bukidnon	Canis lupus familiaris	SEA4-M	OR971619
R10-2021-005	2-Jul-2021	Misamis Oriental	Canis lupus familiaris	SEA4-M	OR971620
R10-2021-006	9-Jun-2021	Misamis Oriental	Canis lupus familiaris	SEA4-M	OR971621
R10-2021-007	7-Jun-2021	Misamis Oriental	Canis lupus familiaris	SEA4-M	OR971622
R10-2021-008	21-May-2021	Misamis Oriental	Canis lupus familiaris	SEA4-M	OR971623
R10-2021-009	18-May-2021	Misamis Oriental	Canis lupus familiaris	SEA4-M	OR971624
R10-2021-010	21-Apr-2021	Misamis Oriental	Canis lupus familiaris	SEA4-M	OR971625
R11-2021-02	1-Jun-2021	Davao del Sur	Canis lupus familiaris	SEA4-M	OR971626
R11-2021-58	10-Jun-2021	Davao del Sur	Felis catus	SEA4-M	OR971627
R11-2021-61	14-Oct-2021	Davao del Norte	Canis lupus familiaris	SEA4-M	OR971628
R11-2021-68	12-Jul-2021	Davao del Sur	Canis lupus familiaris	SEA4-M	OR971629
R11-2022-07	18-Jan-2022	Davao del Sur	Canis lupus familiaris	SEA4-M	OR971630
R11-2022-11	2-Apr-2022	Davao del Sur	Canis lupus familiaris	SEA4-M	OR971631
R11-2022-14	15-Feb-2022	Davao del Norte	Canis lupus familiaris	SEA4-M	OR971632

R11-2022-15	15-Feb-2022	Davao del Norte	Canis lupus familiaris	SEA4-M	OR971633
R11-2022-16	15-Feb-2022	Davao del Sur	Canis lupus familiaris	SEA4-M	OR971634
R11-2022-19	3-Nov-2022	Davao del Sur	Canis lupus familiaris	SEA4-M	OR971635
R11-2022-25	25-Mar-2022	Davao del Sur	Canis lupus familiaris	SEA4-M	OR971636
R11-2022-28	4-Jan-2022	Davao del Norte	Canis lupus familiaris	SEA4-M	OR971637
R11-2022-30	25-Apr-2022	Davao del Norte	Canis lupus familiaris	SEA4-M	OR971638
R11-2022-32	5-Feb-2022	Davao de Oro	Canis lupus familiaris	SEA4-M	OR971639
R11-2022-33	5-May-2022	Davao del Sur	Canis lupus familiaris	SEA4-M	OR971640
R12-2019-0014	9-Jan-2019	Sultan Kudarat	Sus scrofa	SEA4-M	OR971641
R12-2019-0472	7-Jun-2019	Sarangani	Canis lupus familiaris	SEA4-M	OR971642
R12-2019-0521	19-Jun-2019	South Cotabato	Canis lupus familiaris	SEA4-M	OR971643
R12-2019-0538	24-Jun-2019	Sultan Kudarat	Canis lupus familiaris	SEA4-M	OR971644
R12-2019-0611	23-Jul-2019	Sarangani	Canis lupus familiaris	SEA4-M	OR971645
R12-2019-0637	2-Aug-2019	Sultan Kudarat	Canis lupus familiaris	SEA4-M	OR971646
R12-2019-0684	27-Aug-2019	South Cotabato	Felis catus	SEA4-M	OR971647
R12-2019-0699	5-Sep-2019	South Cotabato	Canis lupus familiaris	SEA4-M	OR971648
R12-2019-0861	7-Nov-2019	Cotabato	Canis lupus familiaris	SEA4-M	OR971649
R12-2021-020	5-Jan-2021	Sultan Kudarat	Canis lupus familiaris	SEA4-M	OR971650
R12-2021-033	1-Nov-2021	South Cotabato	Canis lupus familiaris	SEA4-M	OR971651
R12-2021-047	11-Jan-2021	Sultan Kudarat	Canis lupus familiaris	SEA4-M	OR971652
R12-2021-063	11-Aug-2021	Cotabato	Canis lupus familiaris	SEA4-M	OR971653
R12-2021-100	19-Jan-2021	Sultan Kudarat	Canis lupus familiaris	SEA4-M	OR971654
R12-2021-175	2-Feb-2021	North Cotabato	Canis lupus familiaris	SEA4-M	OR971655
R12-2021-176	2-Feb-2021	Cotabato	Canis lupus familiaris	SEA4-M	OR971656
R12-2021-232	16-Feb-2021	South Cotabato	Canis lupus familiaris	SEA4-M	OR971657
R12-2021-237	15-Sep-2021	South Cotabato	Canis lupus familiaris	SEA4-M	OR971658

R12-2021-244	18-Feb-2021	North Cotabato	Canis lupus familiaris	SEA4-M	OR971659
R12-2021-280	23-Feb-2021	Sultan Kudarat	Canis lupus familiaris	SEA4-M	OR971660
R12-2021-339	3-Mar-2021	North Cotabato	Canis lupus familiaris	SEA4-M	OR971661
R12-2021-384	3-Sep-2021	South Cotabato	Canis lupus familiaris	SEA4-M	OR971662
R12-2021-905	24-Jun-2021	North Cotabato	Canis lupus familiaris	SEA4-M	OR971663
R12-2021-954	7-Jan-2021	South Cotabato	Canis lupus familiaris	SEA4-M	OR971664
R12-2021-1086	9-Aug-2021	South Cotabato	Canis lupus familiaris	SEA4-M	OR971665
R12-2021-1087	9-Aug-2021	South Cotabato	Canis lupus familiaris	SEA4-M	OR971666
R13-2021-617	3-Oct-2021	Butuan	Canis lupus familiaris	SEA4-M	OR971667
R13-2021-1498	30-May-2021	Butuan	Canis lupus familiaris	SEA4-M	OR971668
R13-2021-1745	30-Jun-2021	Butuan	Canis lupus familiaris	SEA4-M	OR971669
R13-2021-1748	7-May-2021	Butuan	Canis lupus familiaris	SEA4-M	OR971670
R13-2021-2121	25-Aug-2021	Butuan	Canis lupus familiaris	SEA4-M	OR971671
R13-2021-2131	15-Sep-2021	Agusan Del Sur	Canis lupus familiaris	SEA4-M	OR971672
R13-2021-2136	10-Jun-2021	Butuan	Canis lupus familiaris	SEA4-M	OR971673
R13-2021-2146	18-Oct-2021	Agusan Del Sur	Canis lupus familiaris	SEA4-M	OR971674
R13-2021-2155	20-Oct-2021	Agusan Del Sur	Canis lupus familiaris	SEA4-M	OR971675
