# Supplementary material for: Phylogeography Analysis Reveals Rabies Epidemiology, Evolution, and Transmission in the Philippines
Source: Mol Biol Evol. 2025 Feb 12;42(2):msaf007. doi: 10.1093/molbev/msaf007 (PMC11815495; doi:10.1093/molbev/msaf007)
Supplement: msaf007_Supplementary_Data [file msaf007_supplementary_data.zip › Supplementary Table 4.pdf]

Supplementary table S4. Bayes factors and posterior probabilities of RABV transmission event in different regions of the Philippines.

From	To	Bayes factor	Posterior Probability
CAR	R1	59819.84777	1
CAR	R2	59819.84777	1
NCR	R3	59819.84777	1
NCR	R4A	59819.84777	1
R1	R3	59819.84777	1
R10	R12	59819.84777	1
R3	R4B	2386.413127	0.997222531
CAR	R4A	252.3425036	0.974336185
R10	R13	232.6593279	0.972225308
R10	R9	85.96402272	0.928230197
R2	R3	67.85707929	0.91078769
R6	R7	67.48779191	0.910343295
R6	R8	41.09995393	0.860793245
R3	R4A	20.32652176	0.753582935
R10	R11	18.19823996	0.73247417
R3	R5	16.63214185	0.714476169
R4B	R6	12.41851737	0.65137207
R10	R8	10.3350404	0.608599045
R11	R13	5.895592265	0.470058882
R3	R6	3.356108051	0.335518276
