# Supplementary material for: Phylogeography Analysis Reveals Rabies Epidemiology, Evolution, and Transmission in the Philippines
Source: Mol Biol Evol. 2025 Feb 12;42(2):msaf007. doi: 10.1093/molbev/msaf007 (PMC11815495; doi:10.1093/molbev/msaf007)
Supplement: msaf007_Supplementary_Data [file msaf007_supplementary_data.zip › Supplementary Table 5.pdf]

Supplementary table S5. Results of the generalized linear mixed effects model.

Variables	Univariate analysis		Multivariate analysis	
	Crude OR (95%CI)	p value	Adjusted OR (95%CI)	p value
Annual mean temperature (5°C) (Bio 1)	1.112 (0.479, 2.582)	0.805		
Mean diurnal range (Mean of monthly (max temp - min temp)) (5°C) (Bio 2)	1.296 (0.519, 3.236)	0.578		
Isothermality (Bio 3)	1.001 (0.981, 1.021)	0.953		
Temperature seasonality (standard deviation ×100) (Bio 4)	0.999 (0.994, 1.005)	0.848		
Max temperature of the warmest month (5°C) (Bio 5)	1.196 (0.596, 2.400)	0.614		
Min temperature of the coldest month (5°C) (Bio 6)	0.965 (0.561, 1.662)	0.898		
Temperature annual range (5°C) (Bio 7)	1.171 (0.664, 2.064)	0.586		
Mean temperature of the wettest quarter (5°C) (Bio 8)	1.565 (0.816, 3.002)	0.178	2.134 (1.119, 4.069)	0.021
Mean temperature of the driest quarter (5°C) (Bio 9)	0.752 (0.471, 1.202)	0.233	—	
Mean temperature of the coldest quarter (5°C) (Bio 10)	1.110 (0.474, 2.596)	0.81		
Mean temperature of the warmest quarter (5°C) (Bio 11)	1.044 (0.546, 1.995)	0.897		
Annual precipitation (100 mm) (Bio 12)	1.000 (0.983, 1.018)	0.958		
Precipitation of the wettest month (100 mm) (Bio 13)	0.973 (0.923, 1.026)	0.309		
Precipitation of the driest month (100 mm) (Bio 14)	1.056 (0.814, 1.370)	0.681		
Precipitation seasonality (Coefficient of variation) (100 mm) (Bio 15)	0.730 (0.409, 1.304)	0.287		
Precipitation of the wettest quarter (100 mm) (Bio 16)	0.990 (0.962, 1.019)	0.511		
Precipitation of the driest quarter (100 mm) (Bio 17)	1.035 (0.958, 1.117)	0.382		
Precipitation of the warmest quarter (100 mm) (Bio 18)	1.003 (0.957, 1.052)	0.891		
Precipitation of the coldest quarter (100 mm) (Bio 19)	0.989 (0.963, 1.017)	0.442		
Elevation (100 m)	1.046 (0.953, 1.148)	0.343		
Percentage of agriculture (5%)	1.133 (1.036, 1.238)	0.006	1.153 (1.056, 1.259)	0.001

Percentage of forest (5%)	0.887 (0.812, 0.969)	0.008	—	
Percentage of urban (5%)	1.010 (0.822, 1.242)	0.925		
Leaf area index	1.001 (0.999, 1.002)	0.5		
NDVI	0.987 (0.975, 1.000)	0.053	—	
GDP per capita (1,000$)	1.097 (0.992, 1.212)	0.071	—	
Population density (1,000 people per km^2)	0.999 (0.906, 1.102)	0.991		
Nightlight index	1.002 (0.934, 1.075)	0.949		
Motorized to healthcare institution (5 hours)	1.026 (1.002, 1.050)	0.034	1.043 (1.020, 1.068)	<0.001
Walking to healthcare institution (5 hours)	1.002 (0.993, 1.011)	0.64		
Rodentia richness	1.034 (0.961, 1.113)	0.369		
Carnivora richness	1.259 (0.877, 1.808)	0.212	—	
Chiroptera richness	1.065 (1.028, 1.102)	<0.001	1.051 (1.017, 1.085)	0.003

The underscore represents the variables entered into the multiple factor model selection.
