# Supplementary material for: Phylogeography Analysis Reveals Rabies Epidemiology, Evolution, and Transmission in the Philippines
Source: Mol Biol Evol. 2025 Feb 12;42(2):msaf007. doi: 10.1093/molbev/msaf007 (PMC11815495; doi:10.1093/molbev/msaf007)
Supplement: msaf007_Supplementary_Data [file msaf007_supplementary_data.zip › Supplementary Table 6.pdf]

Supplementary table S6. The spatial resolution, study duration and source incorporated into the risk assessment model.

Type	Data	Spatial resolution	Study duration	Source of data	Website
Ecoclimatic factors	Climate data (Bio1–Bio19)	0° 25′	1975–2018	WorldClim	https://www.worldclim.org/
Environmental factors	Elevation	1 km	2010	EarthEnv	https://www.earthenv.org/
	Cropland	300 m	2001–2020	European Space Agency	https://maps.elie.ucl.ac.be/
	Forest	300 m	2001–2020	European Space Agency	https://maps.elie.ucl.ac.be/
	Urban	300 m	2001–2020	European Space Agency	https://maps.elie.ucl.ac.be/
	Leaf area index	30 m	1981–2019	Resource and Environment Science and Data Center	https://www.resdc.cn/
	NDVI	1 km	2001–2023	Moderate Resolution Imaging Spectroradiometer	https://modis.gsfc.nasa.gov/
Socioeconomic factors	GDP	1 km	1992–2019	Scientific Data	https://doi.org/10.6084/m9.figshare.17004523.v1/
	Population density	1 km	2000–2020	Worldpop	https://www.worldpop.org/
	Nightlight index	1 km	1992–2019	Scientific Data	https://www.nature.com/articles/s41597-022-01322-5

	Motorized to healthcare institution	1 km	2019	Nature medicine	https://www.nature.com/articles/s41591-020-1059-1
	Walking to healthcare institution	1 km	2019	Nature medicine	https://www.nature.com/articles/s41591-020-1059-1
Biological factors	Rodentia richness	10 km	2018	BiodiversityMapping.org	https://biodiversitymapping.org/
	Carnivora richness	10 km	2018	BiodiversityMapping.org	https://biodiversitymapping.org/
	Chiroptera richness	10 km	2018	BiodiversityMapping.org	https://biodiversitymapping.org/

NDVI: normalized difference vegetation index. GDP: gross domestic product.
