# Supplementary figures and images for: Phylogeography Analysis Reveals Rabies Epidemiology, Evolution, and Transmission in the Philippines
Source: Mol Biol Evol. 2025 Feb 12;42(2):msaf007. doi: 10.1093/molbev/msaf007 (PMC11815495; doi:10.1093/molbev/msaf007)

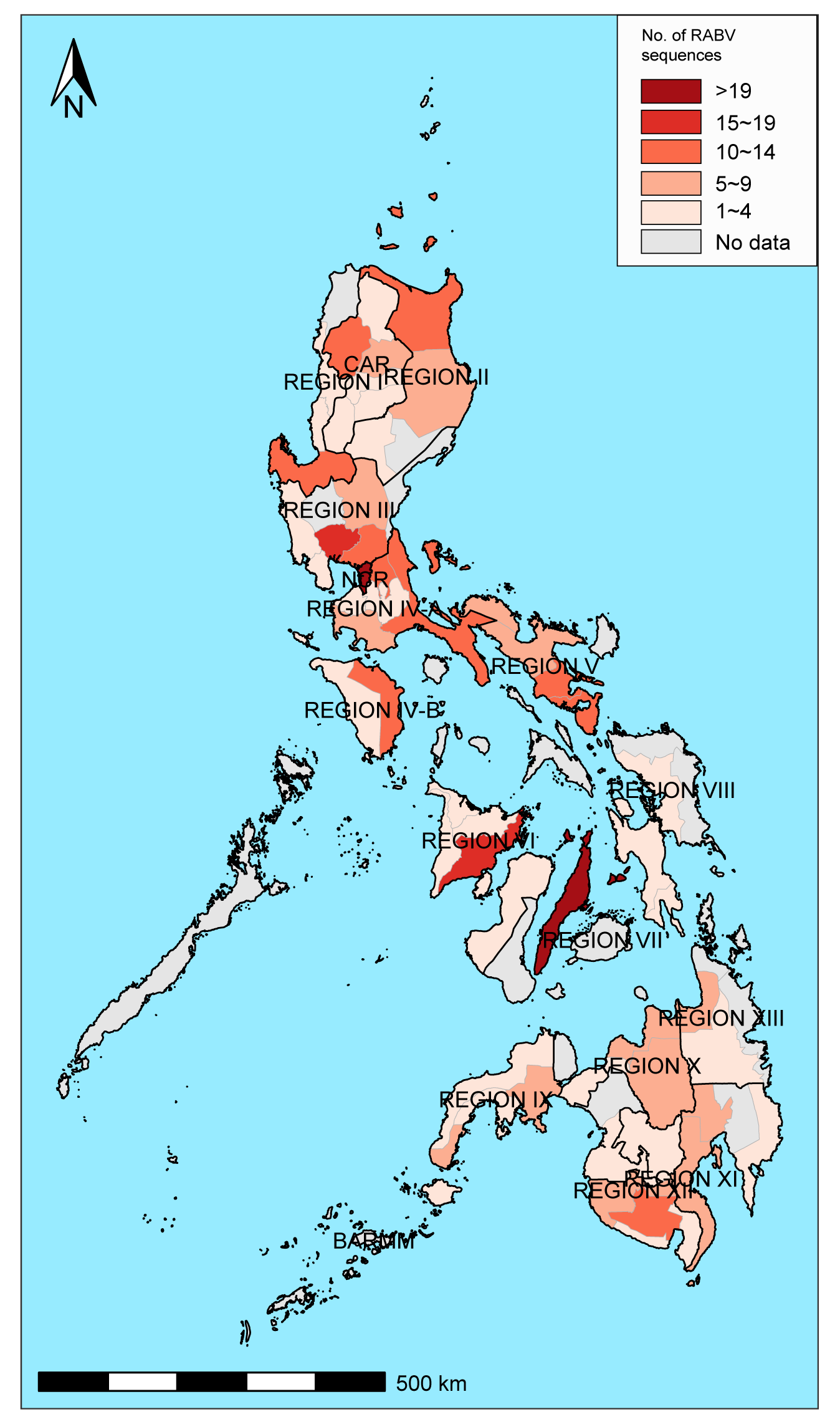

Supplement: msaf007_Supplementary_Data [file msaf007_supplementary_data.zip › Supplementary fig. S1.tif]

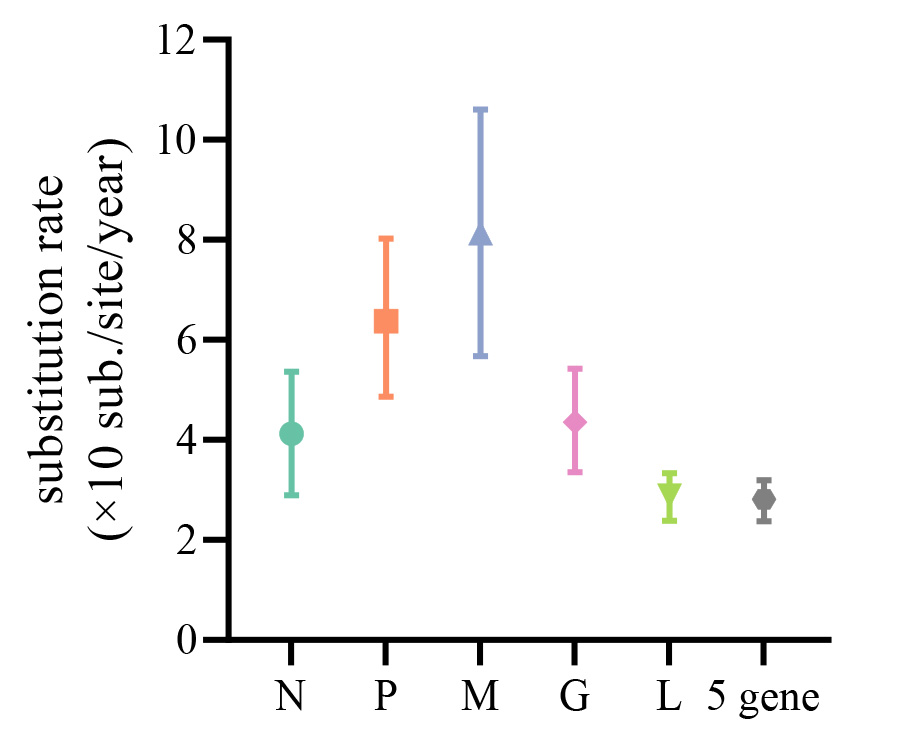

Supplement: msaf007_Supplementary_Data [file msaf007_supplementary_data.zip › Supplementary fig. S2.jpg]
